# The Power to Detect Quantitative Trait Loci Using Resequenced, Experimentally Evolved Populations of Diploid, Sexual Organisms

**DOI:** 10.1093/molbev/msu048

**Published:** 2014-01-18

**Authors:** James G. Baldwin-Brown, Anthony D. Long, Kevin R. Thornton

**Affiliations:** ^1^Department of Ecology and Evolutionary Biology, University of California, Irvine

**Keywords:** simulation, QTL detection, genomics, adaptive evolution, experimental evolution, evolve and resequence

## Abstract

A novel approach for dissecting complex traits is to experimentally evolve laboratory populations under a controlled environment shift, resequence the resulting populations, and identify single nucleotide polymorphisms (SNPs) and/or genomic regions highly diverged in allele frequency. To better understand the power and localization ability of such an evolve and resequence (E&R) approach, we carried out forward-in-time population genetics simulations of 1 Mb genomic regions under a large combination of experimental conditions, then attempted to detect significantly diverged SNPs. Our analysis indicates that the ability to detect differentiation between populations is primarily affected by selection coefficient, population size, number of replicate populations, and number of founding haplotypes. We estimate that E&R studies can detect and localize causative sites with 80% success or greater when the number of founder haplotypes is over 500, experimental populations are replicated at least 25-fold, population size is at least 1,000 diploid individuals, and the selection coefficient on the locus of interest is at least 0.1. More achievable experimental designs (less replicated, fewer founder haplotypes, smaller effective population size, and smaller selection coefficients) can have power of greater than 50% to identify a handful of SNPs of which one is likely causative. Similarly, in cases where *s* ≥ 0.2, less demanding experimental designs can yield high power.

## Introduction

Quantitative traits are of special interest to biologists. The variation in many traits of medical, agricultural, and evolutionary relevance is due to the concerted action of several genes and the environment. Quantitative trait locus (QTL) mapping has been effective at explaining the majority of the heritability of a trait but is poorly suited to resolving the location of QTL beyond several cM (Mackay et al. 2009). More recently, several groups have attempted to increase the resolution of QTL mapping using advanced generation recombinant inbred lines (c.f. Kover et al. 2009; Aylor et al. 2011; King et al. 2012), but resolution is still limited to cM scales. Recently, genome wide association studies (GWAS) have become a major method for investigating the genetic basis for quantitative traits (The Wellcome Trust Case Control Consortium, 2007a, 2007b; Craddock et al. 2010). Although GWAS studies have identified replicable associations between SNPs and complex traits, associated SNPs tend to explain only a small fraction of the heritable variation in the study trait (Manolio et al. 2009), a problem that cannot be solved by increasing sample sizes to tens of thousands of individuals (Signer-Hasler et al. 2012) or replacing SNPchips with complete resequenced genomes (Spencer et al. 2009). Clearly, it is of value to explore novel methods for dissecting complex traits.

In systems that have short generation times and that can easily be reared in the lab in large numbers, an alternative experimental approach to dissecting complex traits has been to “evolve and resequence” (E&R) populations of organisms. E&R studies have been performed with both asexual (Riehle et al. 2001; Barrick et al. 2009; Kishimoto et al. 2010; Tenaillon et al. 2012; Parts et al. 2011) and sexual (Teotónio et al. 2009; Burke et al. 2010; Johansson et al. 2010; Turner et al. 2011; Orozco-Terwengel et al. 2012; Turner and Miller 2012) populations. Because asexual experimental evolution lacks recombination and standing variation in the base population, the footprints of selection in the genome and the means by which an investigator may hope to identify causal variants are different in sexual and asexual systems. Thus, we limit our focus to E&R studies in sexual systems. Under the E&R paradigm, a base population is divided into several replicate populations, half of which are subjected to a well-defined selection pressure, and the other half of which are maintained without selection. Next, the DNA pools from each population are resequenced using NextGen technology and allele frequencies in each pool are estimated. SNPs and/or genomic regions showing consistent differentiation between selected and control population are candidates for harboring causative variants. Studies using this design have claimed to detect numbers of candidate causative sites (CS) from 662 (Burke et al. 2010) to almost 5,000 (Orozco-Terwengel et al. 2012) for various quantitative traits. Currently, the CSs detected by E&R methods have not been validated.

To date, the field of E&R has been almost entirely empirically motivated. Study designs have varied greatly in terms of the number of replicate populations, the population sizes maintained, the number of generations over which the experiment was carried out, and the number of haplotypes in the base population from which selection was initiated. For example, Burke et al. (2010), Teotónio et al. (2009), Turner and Miller (2012), and Orozco-Terwengel et al. (2012) maintained population sizes in excess of 1,000 individuals, whereas Turner et al. (2011) used population sizes of around 225, and Johansson et al. (2010) used effective population sizes of 27 to 44 individuals. The number of founder haplotypes is often not precisely known but can vary from a few dozen individuals (Johansson et al. 2010) to 113 isofemales (Orozco-Terwengel et al. 2012) up to 173 inbred lines (Turner and Miller 2012). The number of generations of evolution also varies widely between experiments: Turner and Miller (2012) used 14 generations of selection, Orozco-Terwengel et al. (2012) used 37, Teotónio et al. (2009) and Johansson et al. (2010) used 50, Turner et al. (2011) used 100, and Burke et al. (2010) used 600. Replication varies as well: Turner and Miller (2012) sequenced two replicate populations each for two experimental treatments, Turner et al. (2011) sequenced two replicate populations each for two experimental treatments and one control, Orozco-Terwengel et al. (2012) sequenced three replicate populations undergoing domestication, Johansson et al. (2010) sequenced two populations selected for divergence, and Teotónio et al. (2009) sequenced 29 total populations—five control populations, four replicate populations for each of three treatments, and four reverse-evolved populations for all three treatments. Burke et al. (2010) sequenced five experimental and five control populations, but each treatment was sequenced as a single pool because of technological constraints. It is of value to quantify the extent to which these experimental design decisions impact the power to detect CSs and contribute to false positives.

Furthermore, there are no agreed upon statistical approaches for analyzing the sets of pooled allele frequency estimates obtained from E&R studies. For example, Burke et al. (2010), Johansson et al. (2010), and Teotónio et al. (2009), respectively, used Fisher's exact test, a χ^2^ test, and an a posteriori Dunnett test to detect significant allele frequency differences between treatments, whereas Orozco-Terwengel et al. (2012) and Turner et al. (2011) used, respectively, the Cochran–Mantel–Haenszel test and a statistic referred to as “DiffStat” to determine whether allele frequencies differed significantly from simulated allele frequencies subject only to drift. Burke et al. (2010) favored sliding windows of allele frequency change. Turner and Miller (2012) used a graphical approach in which the divergence within treatments was used to establish a null expectation, and divergence between treatments was considered significant if it fell outside this null range. The lack of a consistent standard for statistics and experimental conditions prevents us from confirming the numerous candidate CSs that these studies claim to have detected.

The exact prediction of allele frequency change at even a single locus is challenging when both selection and genetic drift affect allele frequency. The approximation of allele frequency probability distribution over time that is best suited to this problem, the Kolmogorov forward diffusion equation (Fisher 1930, reviewed by Kimura 1964), is a second-order partial differential equation that can only be solved by numerical integration in many cases (Gutenkunst et al. 2009). This equation is advantageous in that, unlike the binomial sampling method (Ethier and Nagylaki 1980), it does not make the assumption that Hardy–Weinberg equilibrium is maintained, which is crucial when modeling experimental evolution because of the small population sizes and large selection coefficients involved. The fact that time-dependent diffusion equations often have no closed-form solutions and make the strong assumption of very large population size and weak evolutionary forces (e.g., small s in the case of selection) motivates the use of simulation in this work. To accurately predict the results of E&R experiments without an exact theoretical solution, we chose to quantify the power and false-positive rate of E&R studies via forward-in-time population genetic simulations of evolving 1 Mb regions. We generated base populations with defined numbers of preexisting haplotypes via coalescent simulation (analogous to establishing a laboratory population from a wild caught sample), expanded the base population, and chose diploid individuals to initiate an experimental evolution experiment. Our simulations focused on a single causative SNP, embedded in a 1 Mb region filled with neutral SNPs, under constant selection during the course of the experiment. We designated a single SNP to have a positive selection coefficient in the selected population and a selection coefficient of zero in the control populations and then allowed each population to evolve with selection, recombination, and drift. By simulating replicate E&R studies and then carrying out appropriate statistical tests on the replicated data sets, we obtained an estimate of the proportion of times that a similar experiment would detect a region (CR for causative region) harboring at least one causative SNP and potentially identify a causative SNP (CS) embedded in such a region. Because of the existence of linkage disequilibrium and strong selection during experimental evolution, it may be easier to detect CRs than CSs. We carried out these simulations under a variety of conditions: we varied population size (*n*), number of founder haplotypes (*h*), selection coefficient on the CS of interest (*s*), number of replicated populations (*r*), and number of generations of evolution (*g*) (table 1). We termed these parameter combinations “Θ.” We included control simulations in which the selection coefficient at the causative SNP in the selected population was zero, which allowed us to determine a Type I error rate for CR and CS detection.

**Table 1.** Useful Terms.

	Term	Values Used in Simulation	Description
*r*	Number of replicates	2, 5, 10, 15, 25	The number of independent experimental populations that are used in each trial. There are an equal number of control populations.
*n*	Population size	100, 250, 500, 1,000	The number of diploid individuals that successfully reproduce every generation.
*h*	Number of haplotypes	4, 32, 100, 500	The number of haplotypes present in each population at the start of each experiment. A population originally derived from one male and one female would have four haplotypes.
*g*	Number of generations	100, 500, 1,000	The number of generations of selection that both the control populations and the selected populations have undergone before allele frequency calculation.
*s*	Selection coefficient	0, 0.0005, 0.005, 0.05, 0.1, 0.2	The strength of selection at the causative locus in a particular genomic region.
Θ	Parameter combination		The particular set of *r*, *n*, *h*, *g*, and *s* used in each set of 500 simulations.
MSM	Most significant marker		The SNP that was found to have most significantly diverged in a particular simulation
CS	Causative SNP		The SNP that was selected upon in a particular simulation.
	CR detection power		The fraction of studies of a particular Θ that found at least one significantly diverged SNP
	Exact location power		The fraction of studies of a particular Θ in which the MSM is the CS.
	Within 10 kb power		The fraction of studies of a particular Θ in which the MSM is within 10 kb of the CS.
	Top 25 power		The fraction of studies of a particular Θ in which the CS is one of the 25 most significantly diverged SNPs in the region.
	Within 2 LOD power		The fraction of studies of a particular Θ in which the CS is within a 2 LOD drop of the MSM
	Total Power		The fraction of studies of a particular Θ in which the CR is detected, and the CS is localized according to one of the CS localization methods earlier. In other words, CR detection power * localization power
	MSM-CS distance		The physical distance between the MSM and the CS.
	CS rank		The significance rank of the CS when compared with all other SNPs in the region

We observed that the false-positive rate for CR detection (even when using a very stringent criterion of significance) was extremely high using standard single-marker tests under a minority of conditions when ten replicate populations were used. The power to detect CRs was determined primarily by population size, replication, selection coefficient, and number of generations, with an intermediate number of generations being ideal. The power to localize CSs was similar to the power to detect CRs but was strongly affected by the number of founder haplotypes. Achieving a total power to detect CRs and localize CSs of 80% required almost all parameters to be at their ideal values (1,000 individuals per population, 500 founder haplotypes, and 25 replicate populations) for the case of a selection coefficient of 0.1, but reasonable levels of power can also be achieved with less costly experimental designs or higher selection coefficients. Our simulations suggest that the experimental designs that could be most effectively utilized for detecting CRs and localizing CSs under the E&R paradigm are not currently widely employed and likely require considerable experimental effort. Still, the parameter space that provides reasonable power levels is not outside the realm of possibility for E&R studies using macroscopic organisms.

## Results

### The False-Positive Rate

**Fig. 1.**
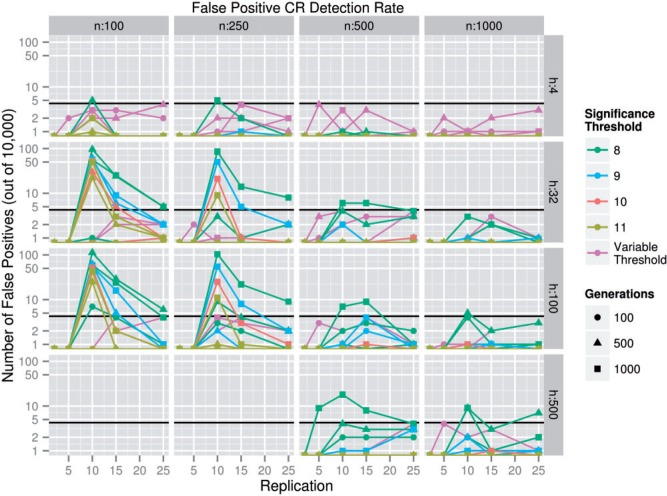
The false-positive CR detection rate versus replication. This plot depicts the fraction out of 10,000 cases in which a region containing no CS contained at least one significantly diverged SNP for four different per SNP –log_10_(*P* value) thresholds. The black line indicates the maximum allowable false-positive rate (4/10,000). *n* represents population size, whereas *h* represents the number of founder haplotypes. The variable significance threshold used in our later power analysis is also included for comparison. When two lines overlap, the line representing a more strict significance threshold is the visible line.

From the perspective of a naïve observer, any given simulated 1 Mb genomic region might or might not contain a CS. To determine the fraction of times that we falsely identified a CR, we calculated for every parameter combination (Θ) where *s* = 0 the fraction of cases in which at least one SNP was found to have a *P* value of less than 10^−^^1^, 10^−^^2^, 10^−^^3^, and so on, through 10^−^^14^. We referred to this as the false-positive CR detection rate (fig. 1); that is, the fraction of neutrally evolving regions that are nonetheless flagged as “significantly diverged.” It is apparent from the figure that the false-positive rate is quite high for certain parameter combinations regardless of the statistical threshold employed. False positives are especially frequent in the specific case in which all the following are true: there are ten experimental replicates, the population size is only 100 individuals, and there are between 32 and 100 founder individuals. This elevated false-positive rate is likely due to the *t*-statistic used to assess significance not being distributed as a *t*-distribution, especially in the tails, when the number of replicates is small (supplementary fig. S1, Supplementary Material online). It is important that our 1 Mb false-positive rate is essentially zero; otherwise, there is a high likelihood of identifying a false-positive CR somewhere in a genome that is several hundred Mb in size. In a genome, the size of *Drosophila melanogaster* (122 Mb), the false-positive CR detection rate necessary to achieve a genome-wide false-positive rate of 0.05 is 0.05/122 = 0.00041. This corresponds to approximately 1 false positive in every 2,439 regions tested. To accurately measure the false-positive rate at low values, we generated 10,000 replicate simulations at each Θ where *s* = 0. With this number of replicate simulations, any Θ with four or fewer false positives has an acceptable error rate. At each Θ, we found the most lenient of our chosen significance thresholds that produced four or fewer false positives and used it in power calculations for the remainder of the experiment (supplementary fig. S2 and table S1, Supplementary Material online). This is a more fair comparison than choosing a single significance threshold that is applied to all Θ because the false-positive rate varies widely between Θ, so that a significance threshold that is reasonable for some Θ is unnecessarily strict for other Θ and would not provide a reasonable estimate of the maximum power achievable in those Θ. Θ, in which an acceptable false-positive rate was not achieved by our most strict significance threshold, 10^−^^14^, were discarded. This includes all experimental designs where *r* = 10, *n* = 100, *h* = 100, and *g* = 500 or 1,000 are simultaneously true. Of the 208 chosen thresholds (one for each combination of *n*, *h*, *r*, and *g*), the distribution was as follows, with the first item in the list corresponding to 10^−^^1^, the second corresponding to 10^−^^2^, and so on: 0, 0, 42, 0, 7, 25, 42, 62, 20, 5, 1, 1, 1, and 2. The large number of significance thresholds set to 10^−^^3^ corresponds to the Θ in which *r* = 2; in these Θ, power and false-positive rates are both extremely low, so the selecting of a lenient significance threshold is unsurprising. The mean of the −log_10_ of the significance thresholds is 6.71.

### Power to Detect a CR as Significant

**Fig. 2.**
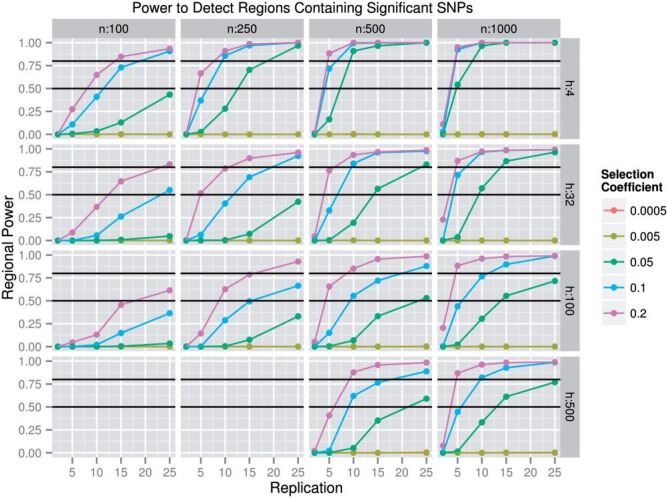
CR detection power. This plot depicts the power to detect regions containing one or more significantly diverged SNPs. The Θ in which all the following are true simultaneously: *r* = 10, *n* = 100, *h* = 100, and *g* = 500 or 1,000 would be omitted due to high false-positive rates, but only *g* = 100 is shown for ease of viewing. *n* represents population size. *h* represents the number of founder haplotypes. The *P*-value threshold for significance was determined for each Θ by finding the most lenient threshold that sufficiently limited false positives. Each point represents 500 independently replicated sets of populations. All lines that are not visible overlap with *s* = 0.005. The black lines indicate power levels of 50% and 80%.

Having controlled the false-positive rate via an individualized statistical threshold, we examined the ability to detect CRs. As in traditional QTL literature, there are two issues at hand. First, is it possible to find an association between genetic features and experimental treatment (this is analogous to CR detection as discussed in this section)? Second, if an association is found, to what level of precision can the polymorphism underlying the trait be localized (this is analogous to CS localization in the following sections)? As above, we considered a CR detected if it contained at least one significantly diverged SNP (*P* ≤ significance threshold). Because we only used 500 simulations per parameter combination where *s* > 0, we estimated the amount of error in estimates of power due to limited sampling by finding the 95% confidence interval around each power estimate using binomial sampling. We found that the mean width of the 95% confidence interval for all nonzero power estimates was 4.64%, the standard deviation of these widths was 3.31%, and the range of widths was 0.052–8.94%. The power to detect a CR increased with increasing *r*, *n*, and *s*, slowly decreased with increasing *h*, and was maximized at *g* = 500 when *s* = 0.05 and at *g* = 100 when *s* ≥ 0.1 (fig. 2). When *r* = 2, we observed a power of near zero in all cases. For several simulated parameter combinations, power was quite high, especially when *n* was large and the *s* associated with the CS was ≥0.05. As expected from standard population genetic theory, as decreasing *s* approached the reciprocal population size, power to detect a CR decreased substantially. Interestingly, although smaller numbers of starting haplotypes are associated with the greatest power to detect a CR, this effect was weak (a feature of E&R experiments that will be important in identifying CSs). Below, we disregarded parameter values where CR power with that parameter value was always below 35%; specifically, we disregarded all Θ where *s* ≤ 0.005, *r* = 2, *n* ≤ 100, or the specific case where *n* ≤ 250 and *r* ≤ 5.

### Power to Identify a CS

**Fig. 3.**
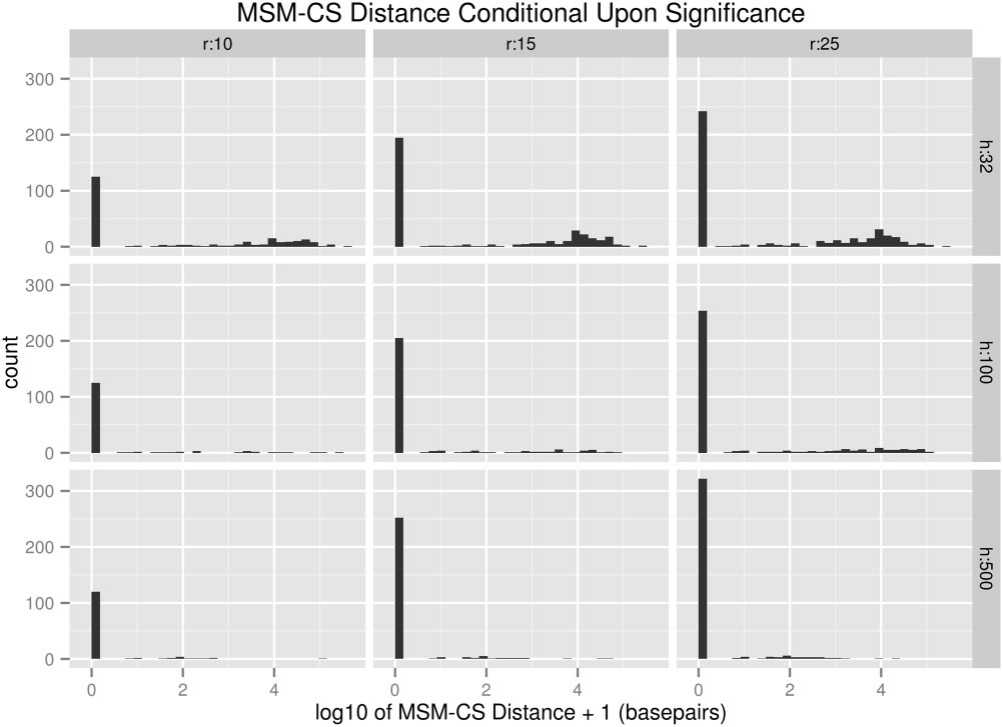
A histogram depicting the distribution of the distance from the MSM to the CS (MSM-CS distance) after 500 generations of selection with 500 individuals per population and a selection coefficient at the CS of 0.05 in all cases where the MSM was significant. Variation in population size is not shown because its effects are similar to variation in replication. The MSM-CS distance is shifted by one base pair, so that MSM-CS distances of 0 are visible after logarithmic transformation. The count refers to the number of pure replicates out of 500 that fell into a given range. Note the increase in low-MSM-CS-distance hits due to selective sweeps when *h* is low.

The goal of an E&R study is CR detection followed by the identification of a CS within the detected CR. To determine most effective method of CS localization, we examined the distance from the most significant marker (MSM) to the causative SNP (MSM-CS distance) in each simulated region in which at least one SNP was significant (fig. 3). A large fraction of MSM-CS distances were equal to zero for cases of Θ where CR detection power was high, indicating that precise localization is possible under some circumstances. The nonzero MSM-CS distances appeared to be skewed, such that a large fraction of MSMs were within 100 kb of the CS, indicating that these MSMs are likely driven to high levels of divergence by linkage to the CS, rather than drift. Indeed, if we take, for example, the (relatively moderately powered, drift-heavy) case in which *s* = 0.05, *n* = 500, *h* = 32, *r* = 10, and *g* = 500, 95% of all nonzero MSM-CS distances were less than 59 kb when only significant regions were considered. For a large portion of the Θ cases with high CR detection power (i.e*.*, *n* = 500, *s* ≥ 0.05, *r* ≥ 10, *h* ≥ 100, except where *n* = 500, *h* = 32, *r* = 10, and *g* = 1,000), the median MSM-CS distance is zero, whereas the mean is a nonzero value. We observed a similar pattern in the CS rank (supplementary fig. S3, Supplementary Material online). Although selective sweeps are clearly visible in the raw significance scores (supplementary fig. S4, Supplementary Material online), the fact that a large majority of the MSMs in most regions with high power have an MSM-CS distance of 0 seems to indicate that a sliding window analysis would be no better than a single-SNP analysis at localizing CSs. Indeed, our attempts to use a sliding window for CS localization by identifying the sliding window with the largest summed −log(*p*) values in each region produced lower power than single-SNP analyses (supplementary fig. S5, Supplementary Material online). Thus, we chose to localize CSs through single-SNP analyses.

**Fig. 4.**
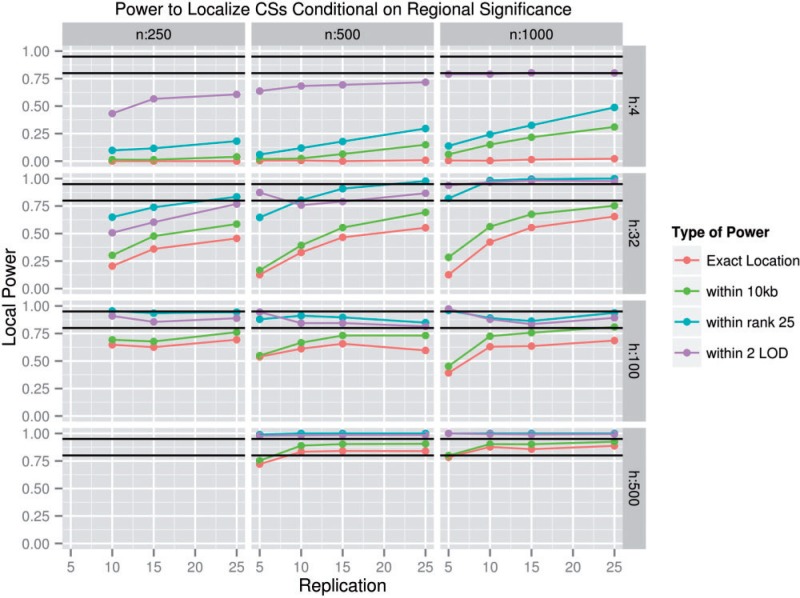
Localization power conditional on regional significance. In other words, the fraction of all significant SNP containing regions in which the CS could be either exactly identified or localized to a small number of candidate SNPs. For clarity, only cases where *s* = 0.1 are shown, but similar patterns occur for *s* = 0.05 and *s* = 0.2. This set of plots shows the fraction of experiments that correctly identified the location of the CS out of all experiments in which at least one SNP was significant. Exact location power refers to cases in which the MSM is the CS, top 25 power refers to cases in which the CS is among the 25 most significant SNPs, within 10 kb power refers to cases in which the MSM is within 10 kb of the CS, and within 2 LOD power refers to cases in which the CS is within 2 LOD of the MSM. The population size is represented by *n*, whereas the number of founder haplotypes is represented by *h*. Nonvisible within 10 kb power points overlap with top 25 power points. The black lines indicate 80% power and 95% power.

We examined several methods of precisely localizing a CS, conditional upon identifying its CR as significant. Most strictly, we may identify a CS as being correctly localized only if the MSM in a CR is the CS. Alternatively, many would consider any analysis that restricts the likely location of a CS to a small region (i.e*.*, 10 kb), a small number of SNPs (i.e*.*, 25), or within a small logarithm (base 10) of odds (LOD) drop of the MSM (i.e*.*, within 2 LOD) to have utility, as additional experiments may be capable of identifying the CS. Figure 4 summarizes the power to localize the CS to: an exact location, within 10 kb of the MSM, within the top 25 most significant SNPs in a region, or within a 2 LOD drop of the MSM, all conditional on CR detection and *s* = 0.1. *g* is set to 500 in all plots below except where specified for ease of viewing, and because the effect of *g* on power was relatively small in the parameter space where power is high. The primary factor that affected CS localization was *h* (the number of founding haplotypes). When *h* is small, it appears that high linkage disequilibrium results in significant allele frequency divergence at SNPs near the CS, making it difficult for the CS to be differentiated from neighboring SNPs. From figure 4, it is apparent that the localization power was quite high provided that *h* was high. *h* negatively affected CR detection power, yet positively affected CS localization power. As discussed later, the overall effect of *h* on power was positive due to the extreme effect of *h* on CS localization power. In a best case scenario where *n* = 1,000, *s* = 0.05, *g* = 500, and *r* = 25, an *h* of four produces an exact location power of only 4.0%, whereas an *h* of 100 produces an exact location power of 76.4%.

The false-positive localization rate, equal to 1 – (localization power), can be considered the fraction of CRs in which the CS is not correctly localized. At least one of the localization false-positive rates calculated is below 5% in 123 of our simulated Θ, including but not limited to the entire simulated parameter space where *h* ≥ 500, *n* ≥ 500, *r* ≥ 10, and *s* ≥ 0.05. It is not possible to calculate a genome-wide false-positive localization rate because the number of expected CRs in a genome is unknown. Note that this false-positive rate is distinct from the false-positive CR detection rate. The false-positive CR detection rate indicates specifically the frequency with which CRs are detected where they do not exist, whereas the false-positive localization rate indicates the fraction of the time that a true CR has its CS incorrectly localized. This value may be of special interest to researchers attempting to assess the chance that a significant SNP in a study is likely to be a CS or merely a neighbor of a CS.

### Total Power

**Fig. 5.**
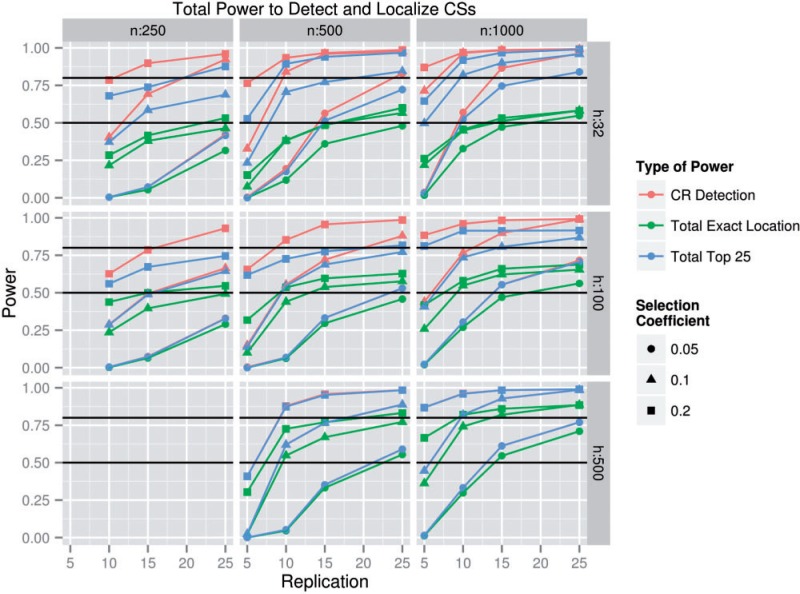
Total power to detect and localize CSs. The ability to detect a CS-containing region and either correctly identify the exact location of a CS or decrease the number of candidate loci to a manageable number after 1,000 generations with a selection coefficient at the CS of 0.05, 0.1, or 0.2. In other words, the fraction of all simulations in which a region contains a significant SNP and one of two methods of detecting a CS is successful: the MSM is the CS (total exact location power) or the CS is one of the 25 most significantly diverged SNPs in the region (total top 25 power). Also shown is the CR detection power, which is the fraction of regions that contained at least one significant SNP. Other measurements of power are excluded for clarity. By design, all total powers listed here must be lower than the CR detection power. The black lines indicate 50% and 80% power. Where CR detection power is not visible on the plot, it overlaps with total top 25 power.

Total power is the product of CR detection power and CS localization power given CR detection. Total power is then the fraction of all CSs that, starting from no prior knowledge about the data, can be detected and localized successfully. Figure 5 gives the total exact power, total top 25 power, and CR detection power as functions of Θ when *g* = 100. The range where *h* = 4 is excluded because the CS localization power conditional upon CR detection in these Θ is less than 80% for all statistics except the within 2 LOD power, and few E&R experiments use only four founder haplotypes. Total power is highest when *s*, *n*, *h*, and *r* are maximized, and *g* is at a value of 100. The parameters necessary to achieve at least 80% exact location power for the *s* = 0.1 case are *n* ≥ 1,000, *r* ≥ 25, and *h* ≥ 500 (fig. 5, supplementary fig. S6, Supplementary Material online). This is a sobering result because it is experimentally difficult (in a system like *Drosophila*) to achieve values of Θ that reach a total exact location power above 80%. On the other hand, in the cases where *s* ≥ 0.1, the same goal of 80% exact location power is much more achievable: 21 of our simulated Θ, including but not limited to all cases in which *s* ≥ 0.1, *h* ≥ 500, *r* ≥ 15, *n* ≥ 1,000, and 100 ≤ *g* ≤ 500 produce a total exact location power greater than 80%. Thus, exact localization requires relatively strict experimental conditions, but strongly selected SNPs are more easily localized. Unsurprisingly, within 10 kb power, top 25 power, and within 2 LOD power were consistently higher than exact location power and were higher than 80% when *s* ≥ 0.05, *n* ≥ 1,000, *r* ≥ 25, *g* = 500, and *h* ≥ 32, except in the case where *s* = 0.05 and *h* = 100, suggesting that ambitious, yet achievable, experimental designs are capable of localizing CSs to a few dozen or even fewer candidate SNPs.

In many experimental systems, there is a direct tradeoff between *n* and *r* when setting up an E&R study because both of these parameters are space and resource limited. Both affect the total power differently: increasing replication (*r)* improves the number of degrees of freedom during statistical analysis, whereas increasing the population size maintained during the experiment (*n)* decreases the effect of genetic drift on allele frequencies. Both parameters are subject to diminishing returns as their values are increased. For example, in the case where *s* = 0.05, *g* = 500, *r* = 10, and *h* = 100, a doubling of *n* from 250 to 500 increases exact location power from 5% to 27%, whereas a doubling of *n* from 500 to 1,000 increases exact location power from 27% to 46%. With the same parameters and an *n* of 500, an increase of *r* from 5 to 10 increases exact location power from 8% to 27%, but a similar increase in *r* from 10 to 15 only increases exact location power from 27% to 43%. Because diminishing returns occur, the ideal *r* and *n* values for a given laboratory size should be balanced, with the specific values depending on the specific conditions of the experiment (supplementary fig. S7, Supplementary Material online). Unfortunately, it is difficult to determine an ideal *r*:*n* ratio because multiple costs are involved: the cost of more replicates versus more generations, the cost of sequencing versus rearing, and so on.

**Fig. 6.**
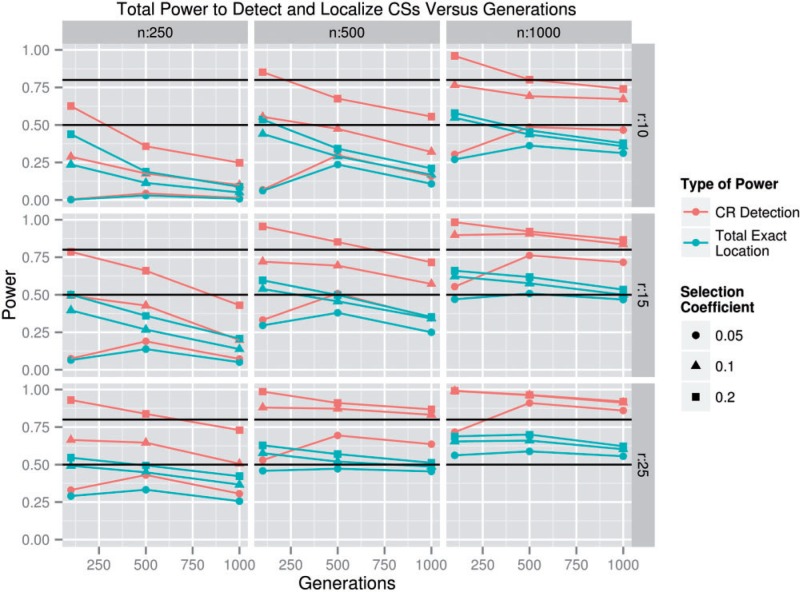
The total power to detect and localize SNPs when *s* ≥ 0.05 and *h* = 100 versus the number of generations of selection. For simplicity, only CR detection power and total exact location power are shown. Variation in *h* is not shown because there are no visible interactions between *h* and *g*. The black lines indicate 50% and 80% power.

**Fig. 7.**
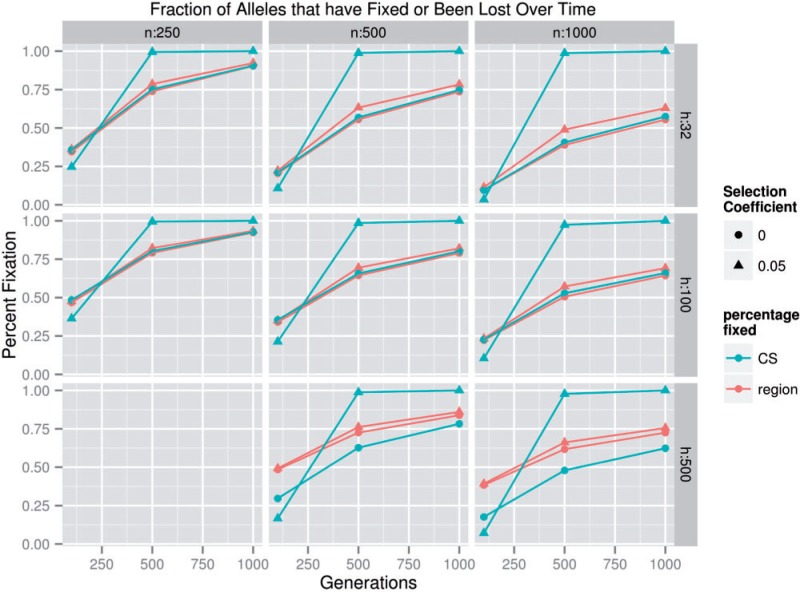
The fraction of alleles that have fixed versus number of generations of selection. The blue line indicates the fraction of all CS alleles that reached an allele frequency of 1 or 0, whereas the red line indicates the fraction of all alleles in the region that reached an allele frequency of 1 or 0. Note that this plot makes use of all available replicates for every Θ. Circles represent *s* = 0, whereas triangles represent *s* = 0.05. Regions with an *s* of 0 have no CS, but the fixation frequency of the centermost SNP is included (the blue lines) for comparison with the CS when *s* = 0.05.

As noted earlier, the effect of *g* on the total power to detect and localize CSs was small in the parameter space where power was high, so *g* was omitted from several plots for simplicity. It was apparent that there was a strong interaction between *g* and *s* with respect to power. At *s* = 0.05, an intermediate *g* (500) appeared to be superior to either high (1,000) or low (100) *g* values in terms of the power to detect CS-containing regions and the total power to localize CSs (fig. 6); at *s* = 0.1 and *s* = 0.2, the relationship between *g* and power was generally negative. One possible explanation for this result is that, when *s* = 0.05, selection had largely fixed any CS’s by generation 500, but drift continued to influence allele frequencies at linked markers past generation 500 resulting in increased noise after 500 generations, whereas CSs with higher selection coefficients, that is, *s* = 0.1 or 0.2, were mostly fixed by generation 100, causing power to decrease when *s* > 100 due to genetic drift. We found that the number of fixed or lost CS alleles in populations where *s* = 0.05 increased from approximately 25% fixed or lost when *g* = 100 up to approximately 100% fixed or lost when *g* = 500 (fig. 7; see supplementary fig. S8, Supplementary Material online, for allele frequencies), but that the total number of fixed alleles continued to increase even when *g* = 1,000, implying that functional standing genetic variation in fitness was largely exhausted by generation 500, but that drift at linked neutral markers continued to occur. This result seems to confirm that rapid selection and slow drift cause intermediate numbers of generations to be ideal for CS detection and localization.

We used multiple linear regression to attempt to create a model that predicts exact location power as a function of the *s*, *r*, *g*, *h*, and *n* (supplementary table S2 and fig. S9, Supplementary Material online). We generated a table of total exact location power and the five experimental design variables of interest, then censored it in R to only contain the Θ where 10 ≤ *r* ≤ 25, 250 ≤ *n* ≤ 1,000, 32 ≤ *h* ≤ 500, 0.05 ≤ *s* ≤ 0.2, and 100 ≤ *g* ≤ 1,000 to focus on modeling the power curve in the area where power is highest. We then used the lm function in R to fit the linear model below:

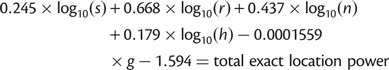



Before calculating the slopes, we modified *s*, *r*, *h*, and *n* by applying the log_10_() function to them as this improved the fit of the model. In the limited parameter space examined, the linear model explains 86.9% (adjusted *R*^2^) of the variation in total exact location power and has a standard error of 0.07782. Values produced by this formula that are above 1 or below 0 should be assumed to be, respectively, 1 or 0. Although this equation does not take interactions between experimental conditions into account, it produces a relatively accurate power estimate in the aforementioned parameter range.

### Multiple Causative SNPs

We simulated the possible case of a 20 Mb chromosome containing 6, 26, or 51 CSs to test the effect of multiple CSs on CR detection power and total power. Specifically, we simulated *s* = 0.05, 0.1, and 0.2 (the selection coefficients that produced reasonable power levels in the previous simulation), *g* = 100, 500, and 1,000, and *r* = 2, 5, 10, 15, and 25. We simulated two different combinations of *h* and *n*: 1) the highest power level that we simulated (*n* = 1,000, *h* = 500) and 2) a moderate power level (*n* = 500, *h* = 100). We generated 250 replicate experiments under each of these parameter combinations. In each replicate, one of the CSs was placed at the center of the chromosome, and the others were randomly distributed throughout the chromosome but not within the 1 Mb region surrounding the central CS. The external CSs always had the same *s* as the central CS. Over the course of the forward simulation, the allele frequencies of all SNPs in the 1 Mb region surrounding the central CS were recorded and used to calculate *P* values. We analyzed the resulting *P* values according to the same framework used in the previous simulation. When compared with the single CS simulation, the multiple CS simulations almost universally produced higher CR detection power and lower CS localization power (supplementary fig. S10, Supplementary Material online). This result is expected. A larger number of neighboring CSs should increase the average significance of SNPs in the region of interest by increasing the probability that an external CS is adjacent to the focal region. This should increase CR detection power by increasing the probability that at least one SNP will be significant, but decrease total power by decreasing the probability that the CS will be the most significant SNP in the region. For the three cases where the number of external CSs was equal to 5, 25, and 50, the average shortest distances from the focal CS to the closest external CS were, respectively, 2.05 Mb, 0.87 Mb, and 0.68 Mb.

One consequence of an increased number of CSs coexisting in a population is an increase in the variance of that population’s fitness. We calculated the fitness of each possible haplotype in each population and found its corresponding frequency in the population to find a distribution (supplementary fig. S11, Supplementary Material online) of fitnesses at each simulated Θ in which more than one CS is present. As expected, the distribution of fitness becomes broader as *s* and the number of CSs increase. The variances that we observed (supplementary fig. S12, Supplementary Material online) when the number of external CSs was 25 or larger seem much higher than those observed in natural populations (Endler 1986, p. 207) and likely much higher than those observed in laboratory experimental evolution (cf. ovary weight in Roff and Fairbairn 2007), indicating that the presence of a large number of CSs with large *s* values is not realistic under these conditions.

## Discussion

This study provides insight into the experimental designs and genome-wide significance thresholds necessary to detect CRs and localize CSs in E&R studies. Importantly, the experimental parameters necessary for CS detection and localization are more difficult to achieve than most experimentalists likely imagine. Researchers wishing to detect more than 80% of the CRs in which *s* = 0.05 are advised to have *r* ≥ 25, *n* ≥ 1,000, and *g* ≥ 500. Researchers wishing to successfully both detect and localize more than 80% of CSs should have *s* ≥ 0.01, *n* ≥ 1,000, *r* ≥ 25, and *h* ≥ 500. No value of *n* simulated in this study was large enough to allow for detection of a useful number of CRs where *s* ≤ 0.005. The low power to detect CSs with small fitness effects is important if we consider that many traits of interest in E&R experiments are quantitative and may have many loci of small effect contributing to standing variation. We have shown that CR detection and CS localization are both improved by high values of *n*, *r*, and *s*. A low *h* value improved CR detection but negatively affected localization, likely because high linkage disequilibrium limited our ability to distinguish between neighboring SNPs. We found that intermediate *g* values provided the highest CR detection and CS localization because most detectable CSs have reached fixation by generation 500 or generation 100 in cases where *s* ≥ 0.1. The large effects that *n*, *h*, and *r* have on power seem to indicate that the most efficient method to increase power is to increase these parameters, especially *r*, which seems to increase total power at a nearly linear rate, at the expense of *g*, which appears to have a small effect on power when other conditions, such as replication, are kept high.

Although the Θ required for very high power is difficult to achieve in practice, we find evidence that reasonable power levels can be achieved fairly easily. For instance, the parameter space in which the total top 25 power was over 50% was quite large (176 Θ). Indeed, all Θ in which *s* ≥ 0.05, *h* ≥ 32, *r* ≥ 15, and *n* ≥ 1,000 produced at least this power level, as did numerous other Θ, such as the Θ where *s* ≥ 0.2, *h* ≥ 32, *r* ≥ 5, *n* ≥ 500, and *g* = 100, except when *h* = *n* = 500, *s* = 0.05, and *r* = 5. These Θ are perhaps more realistically approached than the Θ necessary to achieve 80% total exact location power. This suggests that ambitious but realistic E&R experiments can narrow down CSs to a handful of SNPs in a small genetic region. Such SNPs could be validated via additional experiments such as targeted gene knockout/knockin (Gratz et al. 2013). Given that the primary interest in E&R experiments is to identify the relationship between genotype and phenotype, meaning that validation experiments will be necessary follow-ups to such experiments, we would argue that it is critical to design experiments with high power to localize CSs.

Our results allow us to reflect on the validity of some of the conclusions drawn in published E&R studies by examining the CR detection power and the false-positive rate that we calculated at the Θ that most closely match published experiments. Supplementary figure S13, Supplementary Material online, shows the CR detection power and false-positive rate when *s* = 0.05 at the simulated Θ that most closely match the Θ used by existing studies. The power levels at our chosen significance thresholds and when *s* = 0.05, conditional on using our modified *t*-statistic, were 19%, 1.4%, 0, 0, 0, and 0 for Burke et al. (2010), Teotónio et al. (2009), Johansson et al. (2010), Turner et al. (2011), Turner and Miller (2012), and Orozco-Terwengel et al. (2012), respectively. Respective powers for *s* = 0.1, generated using our chosen significance thresholds, were 27.8%, 44.6%, 0, 0, 0.2%, and 0.2%, whereas respective powers for *s* = 0.2 were 34%, 87%, 0, 0, 20.4%, and 20.4%. Respective significance thresholds chosen by our system (see “The False-Positive Rate” in Results) were 10^−^^7^, 10^−^^6^, 10^−^^3^, 10^−^^3^, 10^−^^3^, and 10^−^^3^. For the same studies, we estimate that CR detection false-positive rates were all equal to 0, again conditional upon our statistical test. Although we estimate a high CR detection rate for Teotónio et al. (2009), the said study genotyped only 55 loci. The odds that any of those 55 loci happened to be close enough to causative SNPs to generate a detectable signal of selection are likely low. Therefore, it may not be reasonable to conclude that Teotónio et al. (2009) is more likely than other articles to have produced a true positive result. It should be noted that several of these studies (Burke et al. 2010; Orozco-Terwengel et al. 2012) claim that all the SNPs that have been detected as significant should be treated as candidate CSs, to the extent that Burke et al. (2010) claim that they have detected, on average, a candidate CS every 175 bp. Given that our simulation shows that it is often more difficult to precisely localize a CS than to detect a CS-containing region, and that SNPs up to 100 kb away from a CS can be brought to significant levels of divergence by said CS, it may be more realistic to say that these studies have detected numerous CRs but have limited ability to precisely localize CSs or to determine the number of CSs present in the genome. Admittedly, all these studies used different test statistics and different significance thresholds than our study, so it is not entirely fair to directly compare the power levels that we estimated from our simulation to the studies in question. That being said, the above studies tended to use a much more aggressive marginal threshold for significance than the ones that we find properly control the false-positive rate. A more fair comparison between this simulation and former studies would require the reanalysis of our simulated allele frequencies and the allele frequency data from each experiment using the statistical methods used by the original investigators. Although this is possible using our simulated data set, it is outside the scope of this investigation.

Despite our simulations suggesting low power and high false-positive rate, several factors prevent the outright dismissal of published studies. First, gene ontology analysis of genes in regions enriched for change in published studies is consistent with the characters being selected upon. For example, the top five enriched gene ontology terms from Burke et al. (2010) were imaginal disc development, smoothened signaling pathway, larval development, wing disc development, and larval development—all have clear causal connections to the “accelerated development” character that was selected. Second, our simulation does not take into account selection coefficients larger than 0.2. Cases of very strong selection on individual CSs could therefore still allow for high power. Even if the majority of the candidate CSs in a given study are false positives, it is still possible that some of them are true CSs. For example, Johansson et al. (2010) examined a small population of artificially selected chickens. Although their *n* of 27–44 should make even the detection of CSs with a selection coefficient of 0.2 difficult in this case, artificial selection usually involves very high selection coefficients that may be high enough to override the force of genetic drift. Johansson et al. (2010) note that, in the candidate QTLs detected in previous studies, estimated selection coefficients (selection against the unfit allele at the candidate QTL of interest) lie in the range of 0.19–0.93, well above the 0.2 simulated here. QTL mapping experiments routinely detect a small number of CSs of relatively large effect (e.g., King et al. 2012), so it follows that under strong selection of the type used in experimental evolution, some CSs should have selection coefficients above 0.05, which could account for Johansson et al.'s (2010) ability (and the ability of other E&R experiments) to detect apparently true CSs. A caveat of this line of reasoning is that routine detection implies selection response is due to a handful of genes of large effect as opposed to dozens to hundreds of genes of much more subtle effect as claimed in the recent E&R literature (Teotónio et al. 2009; Burke et al. 2010; Johansson et al. 2010; Turner et al. 2011; Orozco-Terwengel et al. 2012; Turner and Miller 2012). Thus, the claims of the literature of localization of CSs and dozen to hundreds of sites responding to selection seem mutually exclusive given the experimental designs employed.

This study makes a number of simplifying assumptions, all of which we believe to be realistic when describing the case of experimental evolution of small populations. Our simulation machinery operates based on the Wright–Fisher model of population genetics, in which the gametes of each generation are aggregated into a gene pool to generate the next generation. The assumptions of this model, which generations are discrete and mating is random, are realistic for experimental evolution. A further assumption is that all heritable variation is additive within and between loci. Although it is certainly true that nonadditive variation exists, the majority of heritable variation is likely additive in nature (Hill et al. 2008); therefore, the omitting of nonadditive variation in our power analysis should not dramatically affect our power estimates. Our simulations were limited to 1 Mb gene regions instead of complete genomes, and all simulated regions have one or no selected loci. We detected CRs by determining if a region contained a significant SNP, then localized by identifying the MSM in the region as the CS. Importantly, we observed that the MSM could be quite distant from the CS: even for parameter combinations with high power to detect a region as significant, a small portion of MSMs were up to 100 kb away from the CS, though few were more distant than that (fig. 3). Although our simulations assume that the density of CSs in the genome is relatively low (at most 1 per Mb), our observation that peaks of significant allele frequency change may be quite distant from CSs suggests that the number of significant markers may not be a reliable proxy for the true number of CSs in the genome and call into question whether it is reasonable to deem any significant SNP a candidate CS, especially when *h* is low. In our simulations, when *h* was ≤32 in a population and power was greater than 0, the average exact localization power across our simulations was only 0.244. Although the selection of a 1 Mb region for our simulations was somewhat arbitrary, we believe it is an appropriately sized region to consider. The selective sweeps that occurred in our simulated populations appeared to extend less than 500 kb from the CS under most circumstances (supplementary fig. S4, Supplementary Material online), and few replicate simulations generated an MSM-CS distance greater than 100 kb. Indeed, in the parameter space where *s* ≥ 0.05, 95% of all simulated regions that contained at least one SNP significant at a 10^−^^8^ threshold had an MSM-CS distance ≤ 228 kb. Thus, any SNPs simulated further from the CS would resemble SNPs in neutral regions. Similarly, our decision to use the entire simulated region as a candidate CR instead of using only a limited area (say, 100 kb) is justified in that the various powers simulated here are virtually unchanged when one compares the power using a 1 Mb region and a 100 kb region. The mean difference between the 1 Mb CR detection power and the 100 kb CR detection power is 0.3%, indicating that nearly all CSs that can be detected can be localized to an area the size of a selective sweep around the MSM; in other words, it is reasonable to conclude that CR detection power = total within 100 kb power.

Notably, our goal in simulating 1 Mb regions was not to test the efficacy of the particular CS-detection technique used here; researchers attempting to adapt this technique to empirical use would need to first divide their genome of interest into arbitrary 1 Mb blocks to perform our CR detection step, which would be an unnecessarily arbitrary method of subdividing a genome. Rather, our reasoning for choosing to simulate 1 Mb blocks was to be certain to capture all the genetic change due to linkage to the CS in each simulated region. Our CR detection power is thus an upper bound on the ability of a study with a certain set of experimental parameters to detect the presence of any particular CS. It gives no indication as to the ability of that study to localize that CS, except perhaps to say that if a significant SNP is located, the CS that drove its divergence must be close enough to it to have affected its allele frequency via a selective sweep. Some problematic effects that could occur if our CR detection method were applied as-is to real life data, such as the possibility that a CS could be immediately adjacent to a 1 Mb focal region and could thus drive a SNP to significance in a non-CS-containing region, are not considered further here.

It is possible to imagine much more complicated models and significance tests than the ones we used. For example, we did not attempt to use the combined *P* values of multiple insignificant SNPs to determine the significance of a region because there is no simple way to determine the probability of observing any particular set of multiple *P* values if, as in this case, the *P* values are not independent. Further, the advantage of a combined *P* value approach (higher CR detection) would presumably be at its largest in the Θ where linkage disequilibrium is very high, such as when *h* is low, but such Θ have already been established as having very high CR detection power, so the advantage gained from a combined *P* value approach would be minimal. On a similar note, we did not attempt to simulate a distribution of selection coefficients across the loci in our simulated genomic regions. Recent studies have raised the question as to whether the majority of heritability for any particular trait is best explained by a small number of mutations of large effect (Johnson and Barton 2005) or a large number of small effect mutations (Barton and Turelli 1989; Barton and Keightley 2002). Because there is not a scientific consensus on the question of QTL effect size, and thus selection coefficient, distributions, we chose to avoid making assumptions about selection coefficient distributions, and instead merely simulated a range of selection coefficients and calculated the power to detect CSs at each selection coefficient level. Similarly, we chose not to simulate genomic regions containing multiple CSs because they did not fit the paradigm of this study. The design of this study, in which small genomic regions are simulated, implicitly assumes that CSs are distant enough from each other as to not interact significantly. Were we to relax this assumption, the most appropriate method for simulating multi-CS interactions would be to simulate an entire chromosome and distribute CSs across it. Doing so was outside the scope of this study.

A final model we did not consider is the possibility of plateauing allele frequencies due to diminishing selection pressure as a phenotypic optimum is approached, as hypothesized in Burke et al. (2010) and Burke and Long (2012) (also cf. Sellis et al. 2011), based on a model in Chevin and Hospital (2008) and potentially observed in Orozco-Terwengel et al. (2012). That is, we assumed that immediately following the placement of the selected populations into a novel environment, a previously neutral SNP obtains a new fixed positive selection coefficient. An additive CS that follows a plateauing allele frequency trajectory could be more difficult to detect than one in which allele frequencies approach fixation because of the lower total level of divergence expected in a plateauing allele; however, our simulation indicates that there are diminishing returns on power from increased allele frequency divergence over time (supplementary fig. S8, Supplementary Material online), indicating that plateauing allele frequency trajectories will not severely reduce power. This is evidenced by the fact that, at the Θ where *n* = 1,000; *h* = 500, *r* = 25, and *s* = 0.05, a near doubling of mean CS allele frequency over all 500 simulation replicates from 52% at generation 100 to 94% at generation 500 only increased total exact location power from 71% to 76%.

Our simulation indicates that, in spite of their inability to detect CSs of very small effect, E&R studies should be capable of detecting and localizing the majority of CSs of moderate to large effect under conditions that, whereas more labor-intensive than traditional experimental evolution conditions, are still feasible. The effectiveness of the next generation of E&R experiments will depend on their ability to improve upon the experimental designs of the past by using large, well-replicated, initially diverse populations.

## Materials and Methods

### The Simulation

We simulated replicated experimental evolution using a two-stage approach. First, we simulated 1,000 replicates of a sample of size 2,000 chromosomes from a Wright–Fisher population using the “macs” software (version 0.4b, Chen et al. 2009) using the following parameters: macs 2000 1000000 -t 0.01 -r 0.1 -i 1000 -s $RANDOM. This command line specifies 1,000 replicate simulations of a sample of 2,000 chromosomes. The locus length is 1 million base pairs mutating at rate θ = 4, Nu = 0.01 per site, and recombining at rate *ρ* = 4N*(recombination rate) = 0.1 per site, where *N* is the size of a Wright–Fisher population and *u* is the mutation rate, per base pair per generation. The mutation parameter was chosen to mimic SNP density in non-African *Drosophila melanogaster*, and the recombination rate was based on estimates from Chen et al. (2009).

The outputs from macs were used to seed forward-time simulations using the first *h* haplotypes from a coalescent simulation. The forward-time simulation used here is based on a generic C++ library (Thornton KR, unpublished data) previously used in Thornton et al. (2013). The speed of the library compares favorably to existing forward simulations (Aberer and Stamatakis 2013; Messer 2013) but has the advantage that new models are easily implemented by enabling simple code reuse via the C++ template mechanism. Haplotypes in macs are not sorted, so choosing the first *h* haplotypes is equivalent to randomly choosing *h* haplotypes. These *h* founding haplotypes were replicated *r* times, and then *r* large “base” populations of *n* diploids each were generated by sampling with replacement from the initial coalescent simulations. A single site was assigned a positive selection coefficient, and experimental evolution was simulated using forward-in-time simulations. The forward in time simulations were carried out with various population sizes (*n*), numbers of founder haplotypes (*h*), numbers of replicate populations evolved (*r*), numbers of generations of experimental evolution (*g*), and selection coefficients (*s*). Note that, because of the lack of population structure in these populations, the actual population size should be equal to the effective population size; in a study of real data, the effective population size would be more comparable to the *n* used here because of nonrandom mating and population size fluctuations. The SNP under selection, or the CS, was always the centermost SNP in the region. The selection scheme was codominant with fitnesses 1, 1+*s*/2, and 1+*s*, where *s* is the selection coefficient on the CS. CSs thus followed the same distribution of initial allele frequencies as all other SNPs in the simulation, consistent with a SNP that is initially neutral, but that is selected upon following a change in environmental conditions. Linkage disequilibrium between SNPs is initially an outcome of the neutral Wright–Fisher sampling process used to generate the *h* founder haplotypes, and subsequently determined by the details of the forward-in-time simulation. The forward simulations assume no further mutation in the region, and the recombination rate used was 0.025 per diploid per generation assuming that the 1 Mb region is 5% of a “typical” 20 Mb chromosome whose total recombination rate per generation is 0.5.

To find the experimental parameters best suited to E&R CR detection and CS localization, we arranged our simulated genomic regions as one would arrange a set of populations for experimental evolution. In each replicate simulation, we set up an equal number of experimental and control populations (in which the selection coefficient at the CS is equal to zero), all containing individuals with the same genomic region. Haplotypes were generated based on the initial allele frequencies, and individuals carrying these haplotypes were created. A forward-in-time simulation was then used to keep track of the movement of haplotypes over time with recombination and selection applied. Allele frequencies were calculated and recorded at 100, 500, and 1,000 generations. Although a true E&R experiment would have allele frequency estimation errors that are a complex function of number of individuals sequenced, library preparation methods, average sequence coverage, and variation in sequencing coverage, we chose to simulate the best-case scenario where all allele frequencies are estimated without error. Thus, our estimates of power are likely somewhat optimistic. We performed simulations that varied in population size (*n* diploids), number of founder haplotypes (*h* = twice the number of founding diploids), number of replicate populations (*r*), and selection coefficient (*s*) on the CS. In total, 500 replicate simulations were performed under each of 840 possible combinations of experimental parameters (Θ) (table 1). Combinations in which *h* was larger than *n* were not simulated because such populations would presumably closely resemble populations in which *h* was reduced to the level of *n*. At each number of generations (*g*) in which allele frequencies were recorded, a modified *t*-statistic was calculated on arcsine square-root transformed SNP frequencies using 2, 5, 10, 15, or 25 replicate populations. This empirical Bayesian *t*-statistic (Baldi and Long 2001) indicates the degree to which the allele frequencies of SNPs in the selected populations have diverged from the same allele frequencies in the control populations. It differs from a standard *t*-statistic in that it is not infinity in the case where a SNP of interest is fixed in all experimental replicates and lost in all control replicates. The expression for the modified *t*-statistic is

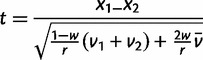

where *x*_1_ and *x*_2_ are the mean allele frequencies across all experimental replicates in selected and control treatments, respectively, *v*_1_ and *v*_2_ are the respective variances, *r* is the number of replicates, and *w* = 0.1. 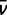 is the average within treatment variance in allele frequency averaged over all SNPs in the region and both treatments. 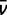 is then an empirically motivated Bayesian prior on allowable variances in allele frequencies and has the effect of stabilizing the denominator of the *t*-statistic. This is especially important in experimental evolution experiments in which a SNP could differentially fix in the experimental versus control replicates purely due to drift alone and thus be associated with a traditional *t*-statistic of infinity.

*P* values were calculated from the modified *t*-statistic using the pt function in R, using a two-tailed method (e.g., see supplementary fig. S4, Supplementary Material online). A 2-tailed *t*-test was used to avoid making a priori assumptions about the nature of the two alleles involved at any given locus: because either allele could be beneficial in theory, it is not reasonable to assume that only the allele whose frequency is being tracked could be beneficial. Degrees of freedom were considered to be 2 *r − *2 (because there are control and experimental treatments, the total number of replicates is twice the number per treatment). The threshold for significance was set independently for each Θ by calculating the false-positive rate for every whole-number power of 10 from 10^−^^1^ to 10^−^^14^ and choosing the most lenient threshold with an acceptable false-positive rate (see “The False-Positive Rate” in Results). Power was calculated by finding the fraction out of 500 times that 1) at least one SNP was significantly diverged in a region of interest and 2) a secondary condition was met. These secondary conditions included having the MSM in the region be the CS (exact location power), having the MSM be within 10 kb of the CS (within 10 kb power), having the CS be among the top 25 MSMs (top 25 power), and having the CS's LOD (logarithm base 10 of odds) score be within two of the MSM's LOD score (within 2 LOD power). The power to fulfill the first condition without regard for a second condition was termed “CR detection power.” This diversity of methods of SNP localization allowed us to determine which method would be most reliable under any particular set of experimental parameters. The CR detection false-positive rate was determined by finding the fraction of cases in which a region with an *s* of zero contained at least one significant SNP. The distance from the MSM to the CS (MSM-CS distance) and the rank of the CS's *P* value compared with the other SNPs in its region (CS rank) were calculated in every replicate of the 500 replicate simulations per Θ to analyze the distribution of significant SNPs across parameter values.

An additional 18,000 replicate genome regions were generated in macs and used to seed forward-in-time simulations with no selection under all of our Θ where *s* = 0. 10,000 replicate neutral simulations were performed for each Θ where *s* = 0. These additional replicates were used to more accurately calculate the false-positive CR detection rate. This high level of replication was only required for the calculation of false-positive rates because the maximum allowable false-positive rate is too small (∼1/2,000) to be accurately measured with only 500 replicate experiments.

### The Data

This simulation produced 2,205,000 semi-independent experimental results. There are 500 pure replicates of each possible permutation of five distinct experimental parameters' values (table 1)—number of replicate populations (*r*), number of haplotypes in the base population (*h*), population size (*n*), selection coefficient at the selected locus (*s*), and number of generations of selection (*g*). The resulting data sets are not completely independent because the coalescent simulation used to generate the 1 Mb regions used here was only run 500 times (10,000 where *s* = 0), and the resulting 500 (or 10,000) genome regions were reused for the 500 (or 10,000) replicate experiments for each parameter combination. Further, for any particular combination of *n*, *r*, *s*, and *h*, the three *g* values simulated were not entirely independent because the data associated with larger *g* values were derived from continuing the forward simulations of the smaller *g* simulations. Values were chosen based on the specifics of the variable: *h*, *n*, and *g* values were chosen based on the levels historically used in experiments of this type. The *r* values were chosen based on the level of replication commonly used in experimental evolution and the level of replication required for high power. Experimental evolution of sexual organisms is usually carried out with five or fewer replicate populations due to the difficulty of rearing large numbers of populations, but, especially in genomics, statistical significance is difficult to achieve with low *r* values because the large number of independent comparisons require a strict significance threshold. *s* values were chosen based on the minimum selection strength necessary for selection to have an effect sufficiently stronger than genetic drift to produce a measurable change in allele frequencies: when the selection coefficient at an SNP is less than ∼1/(2*n*), genetic drift is a more powerful force than selection (Crow and Kimura 1970, p. 425). The *s* values were thus chosen to cover a range of possible sizes, from an *s* considerably smaller than 1/(2*n*) to an *s* larger than 1/(2*n*).

### Data Availability

The simulation code and all data and the necessary code to recreate the data are available online at http://www.molpopgen.org/Data (last accessed February 4, 2014), as is a commented copy of the scripts used to calculate *P* values, power, and other statistics. macs is available at http://code.google.com/p/macs/ (last accessed February 4, 2014).

## Supplementary Material

Supplementary tables S1 and S2 and figures S1–S13 are available at *Molecular Biology and Evolution* online (http://www.mbe.oxfordjournals.org/).

Supplementary Data
