# Supplementary material for: The Power to Detect Quantitative Trait Loci Using Resequenced, Experimentally Evolved Populations of Diploid, Sexual Organisms
Source: Mol Biol Evol. 2014 Jan 18;31(4):1040–55. doi: 10.1093/molbev/msu048 (PMC3969567; doi:10.1093/molbev/msu048)
Supplement: Supplementary Data [file supp_31_4_1040__index.html]

The Power to Detect Quantitative Trait Loci Using Resequenced, Experimentally Evolved Populations of Diploid, Sexual Organisms — The Power to Detect Quantitative Trait Loci Using Resequenced, Experimentally Evolved Populations of Diploid, Sexual Organisms — Supplementary Data 

# The Power to Detect Quantitative Trait Loci Using Resequenced, Experimentally Evolved Populations of Diploid, Sexual Organisms

## Supplementary Data

files

**Files in this Data Supplement:**

- Supplementary Data - pdf file
- Supplementary Data - xlsx file
- Supplementary Data - xlsx file
