# Supplementary material for: The Power to Detect Quantitative Trait Loci Using Resequenced, Experimentally Evolved Populations of Diploid, Sexual Organisms
Source: Mol Biol Evol. 2014 Jan 18;31(4):1040–55. doi: 10.1093/molbev/msu048 (PMC3969567; doi:10.1093/molbev/msu048)
Supplement: Supplementary Data [file supp_msu048_supplementary_figures.pdf]

Supplementary Figure 1

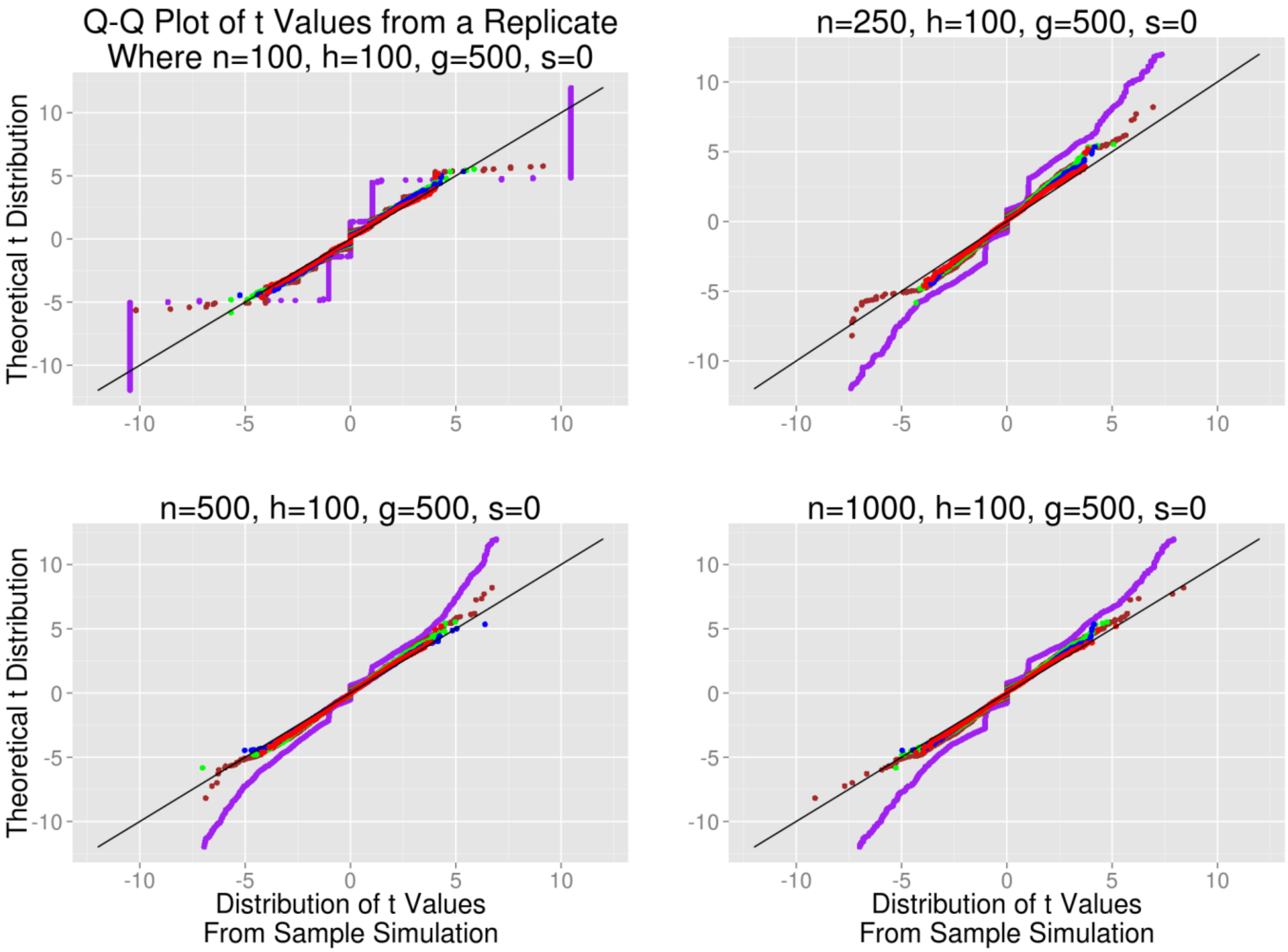

Sup. Fig. 1: This figure depicts a set of Q-Q plots illustrating the distribution of the t statistic used in this study versus a theoretical t distribution. In each case, the t values were calculated from a single, randomly chosen replicate simulation. In all cases, $s = 0$ so that the plots will illustrate the effect of drift alone on the generated t values. Purple, brown, green, blue and red points correspond, respectively, to the cases where $r = 2$, $r = 5$, $r = 10$, $r = 15$, and $r = 25$.

Supplementary Figure 2

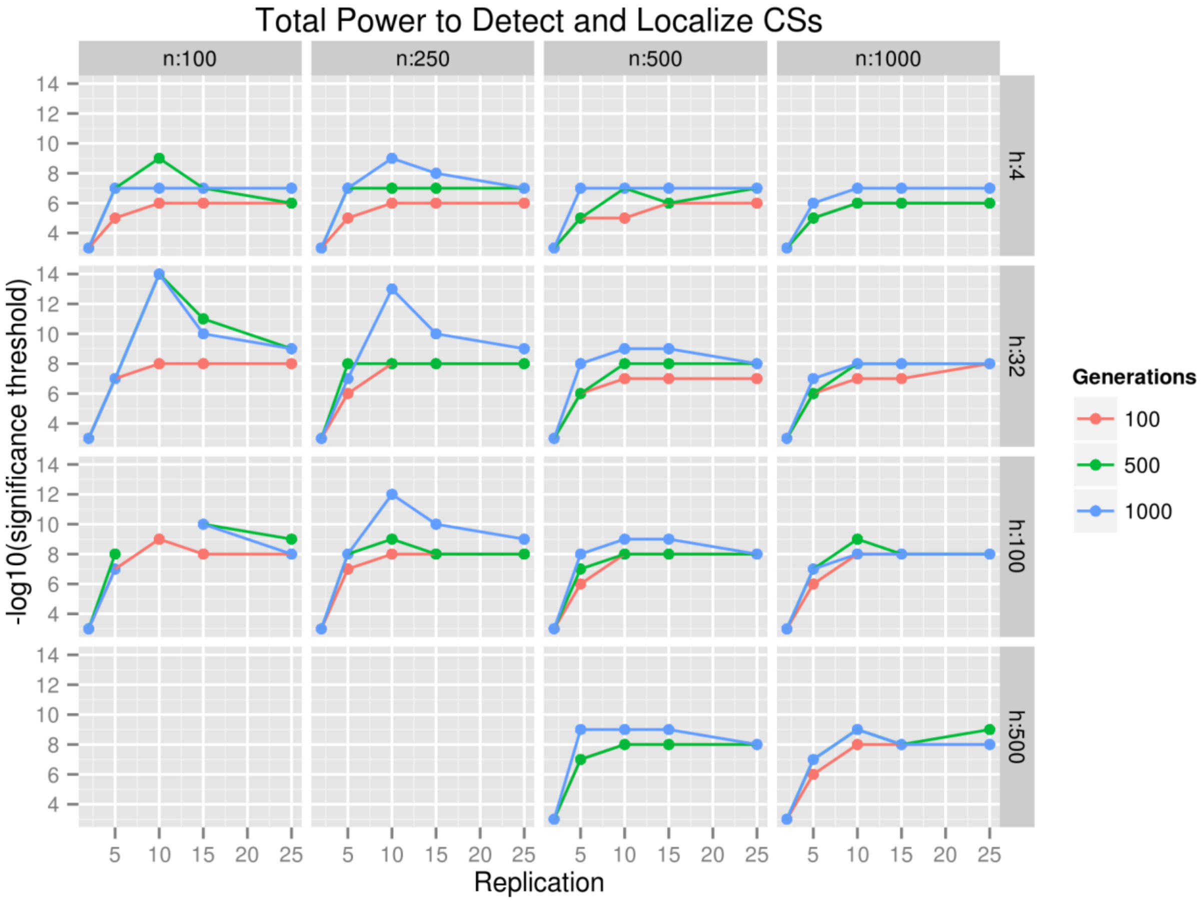

Sup. Fig. 2: The $-\log_{10}(p)$ value chosen as the significance threshold for every simulated parameter combination. Because false positive rates are independent of *s*, *s* is not shown. Parameter combinations that produced unacceptably high false positive rates even when the significance threshold was $p \leq 10^{-14}$ are depicted as missing points.

Supplementary Figure 3

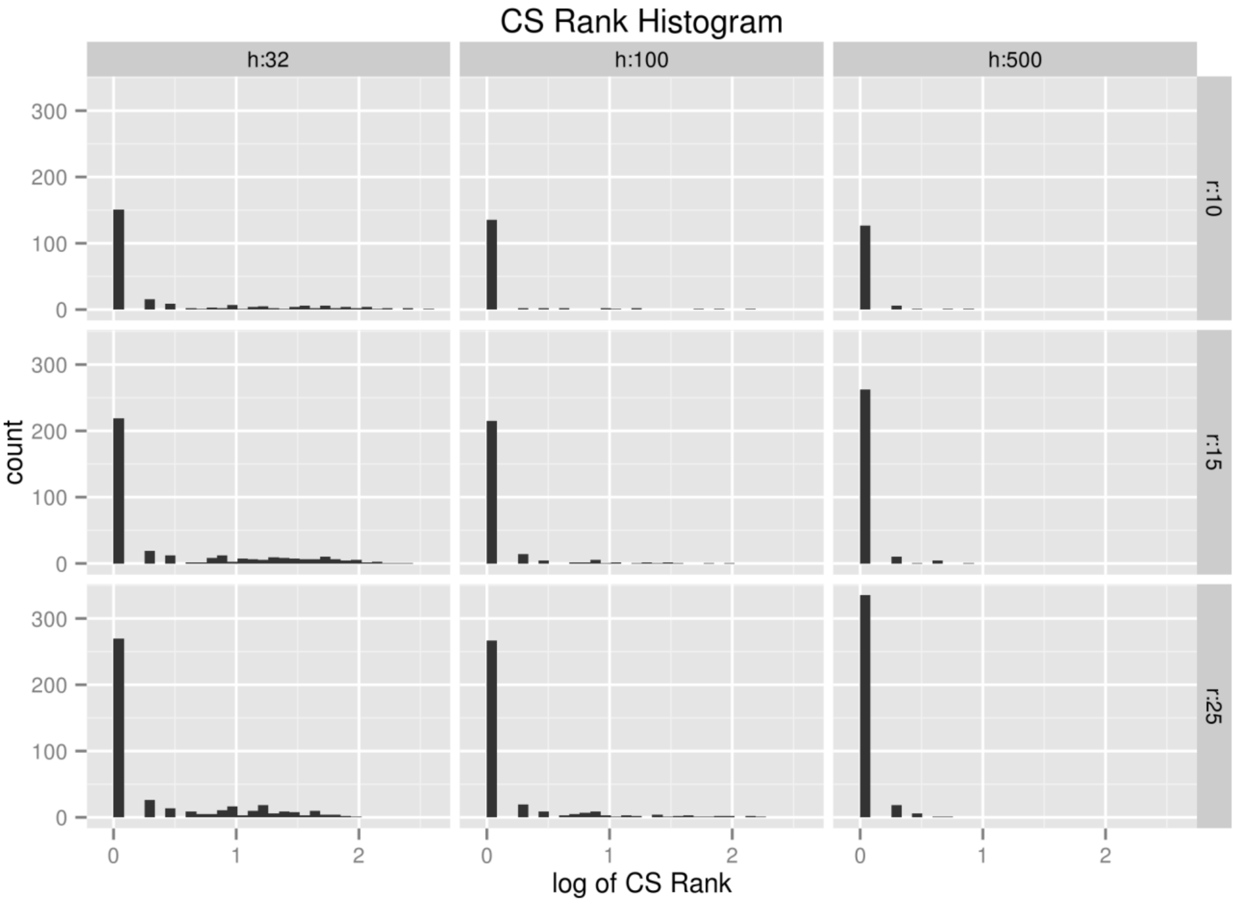

Sup. Fig. 3: A histogram depicting the distribution of the rank order position of the CS when all SNPs in a region are ordered from most significant to least significant after 500 generations of selection with 500 individuals per population and a selection coefficient at the CS of 0.05 in all cases where the MSM was significant. Variation in population size is not shown because its effects are similar to variation in replication. The count refers to the number of pure replicates out of 500 that fell into a given range. Note the increase in low-CS-rank hits due to selective sweeps when haplotype number is low.

Supplementary Figure 4

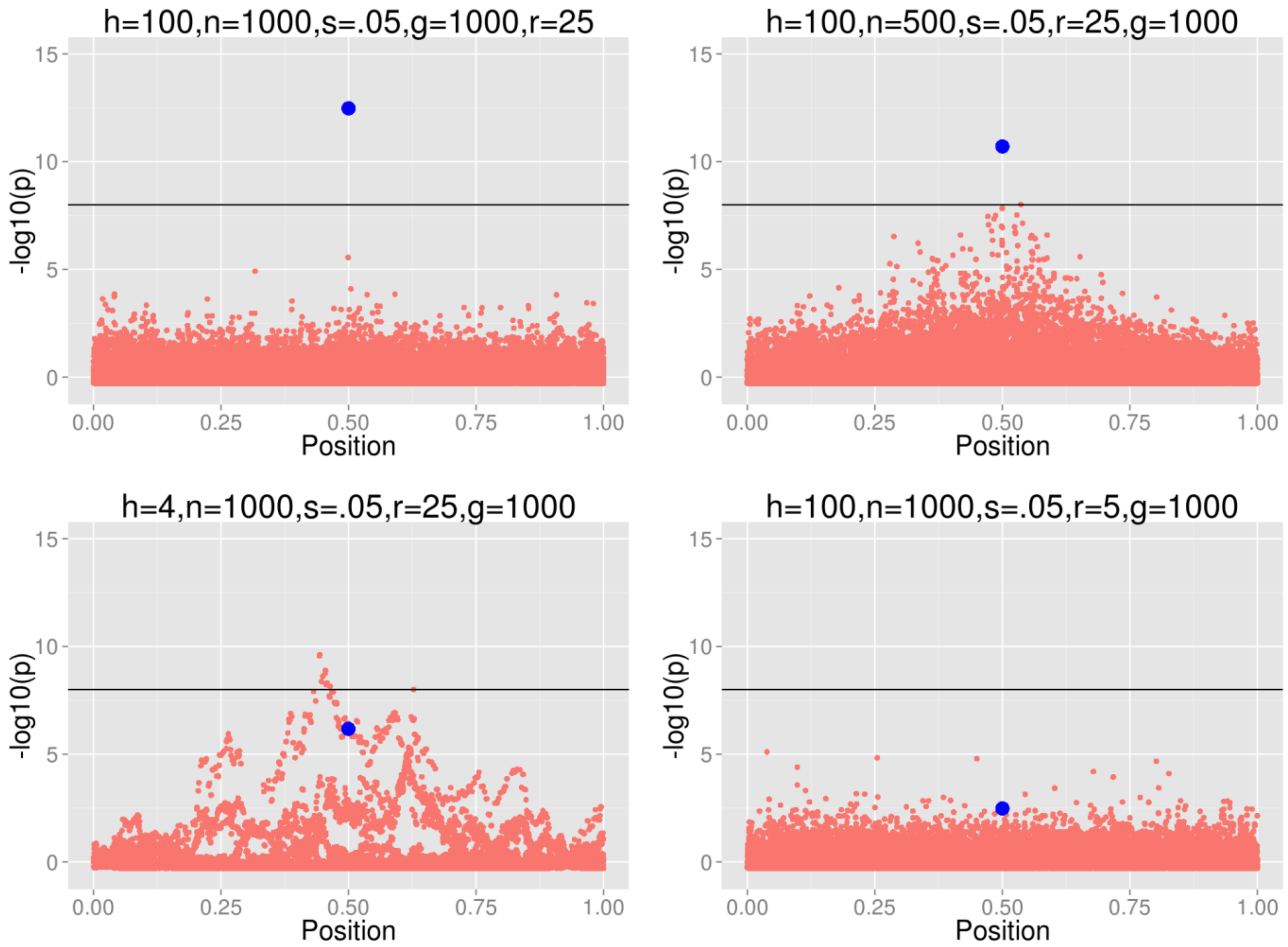

Sup. Fig. 4: Sample plots of significance across genome regions under various Θ. The blue point is the CS, while the red points represent all other SNPs. Top left is a plot of relatively ideal conditions, where the CS is the MSM and no selective sweeps are evident. Top right is a plot showing diminished *n*. Bottom left shows the blocks of linkage disequilibrium found when *h* is low. Bottom right shows the inability to detect the causative SNP when *r* is low.

Supplementary Figure 5

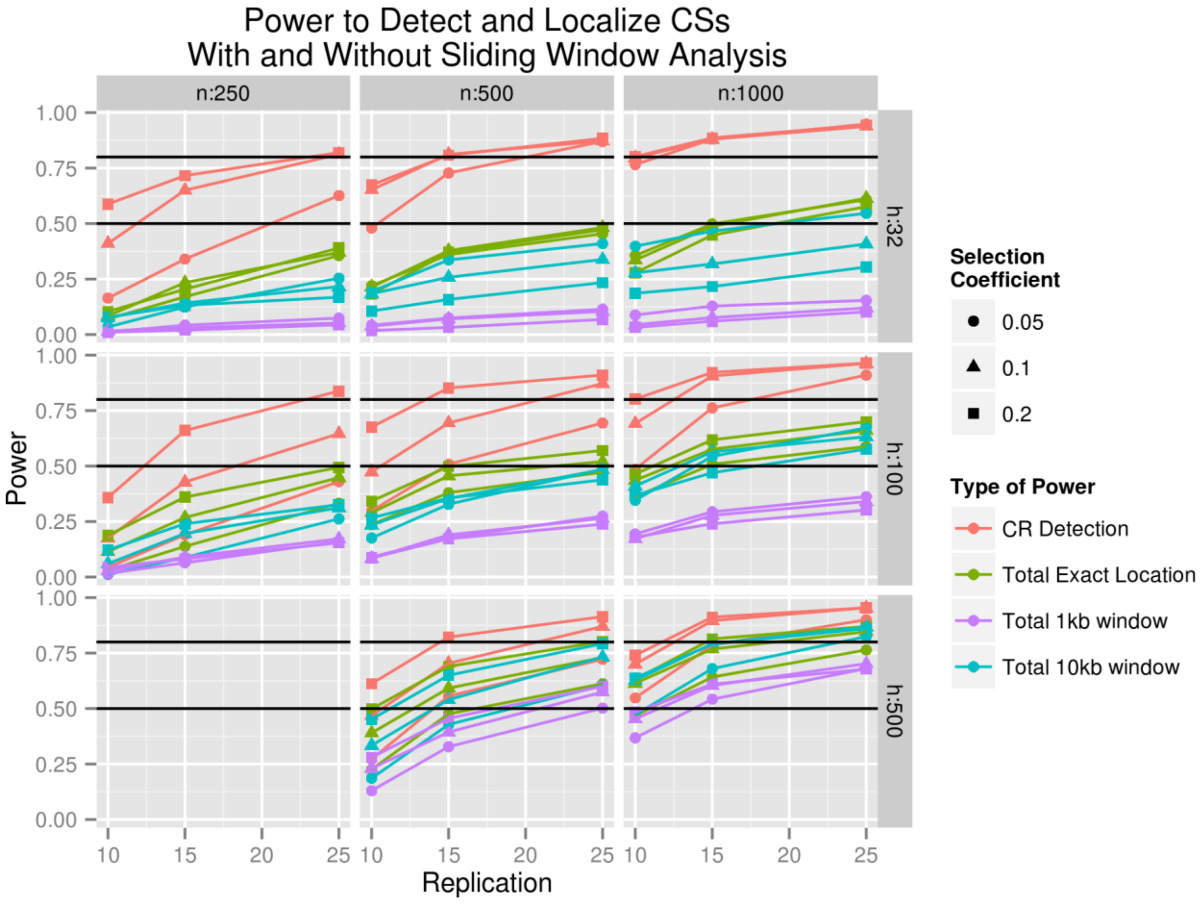

Sup. Fig. 5: The ability (total power) to detect a CS-containing region and either correctly identify the exact location of a CS or decrease the number of candidate loci to a manageable number after 1000 generations with a selection coefficient at the CS of 0.05 or higher. In other words, the fraction of all simulations in which a region is a region contains a significant SNP and one of three methods of detecting a CS is successful: the MSM is the CS (Total Exact Location Power), the CS is included in the most significant 1kb window in the region (Total 1kb window power) or the CS is included in the most significant 10kb window in the region (Total 10kb window power). Also shown is the CR Detection Power, which is the fraction of regions that contained at least one significant SNP. By design, all total powers listed here must be lower than the CR detection power. In every instance where at least one SNP was significant, the window with the largest sum of log10(p) values was found. If the most significant window in the region contained the CS, the CS was considered correctly detected. The black lines indicate 50% power and 80% power.

Supplementary Figure 6

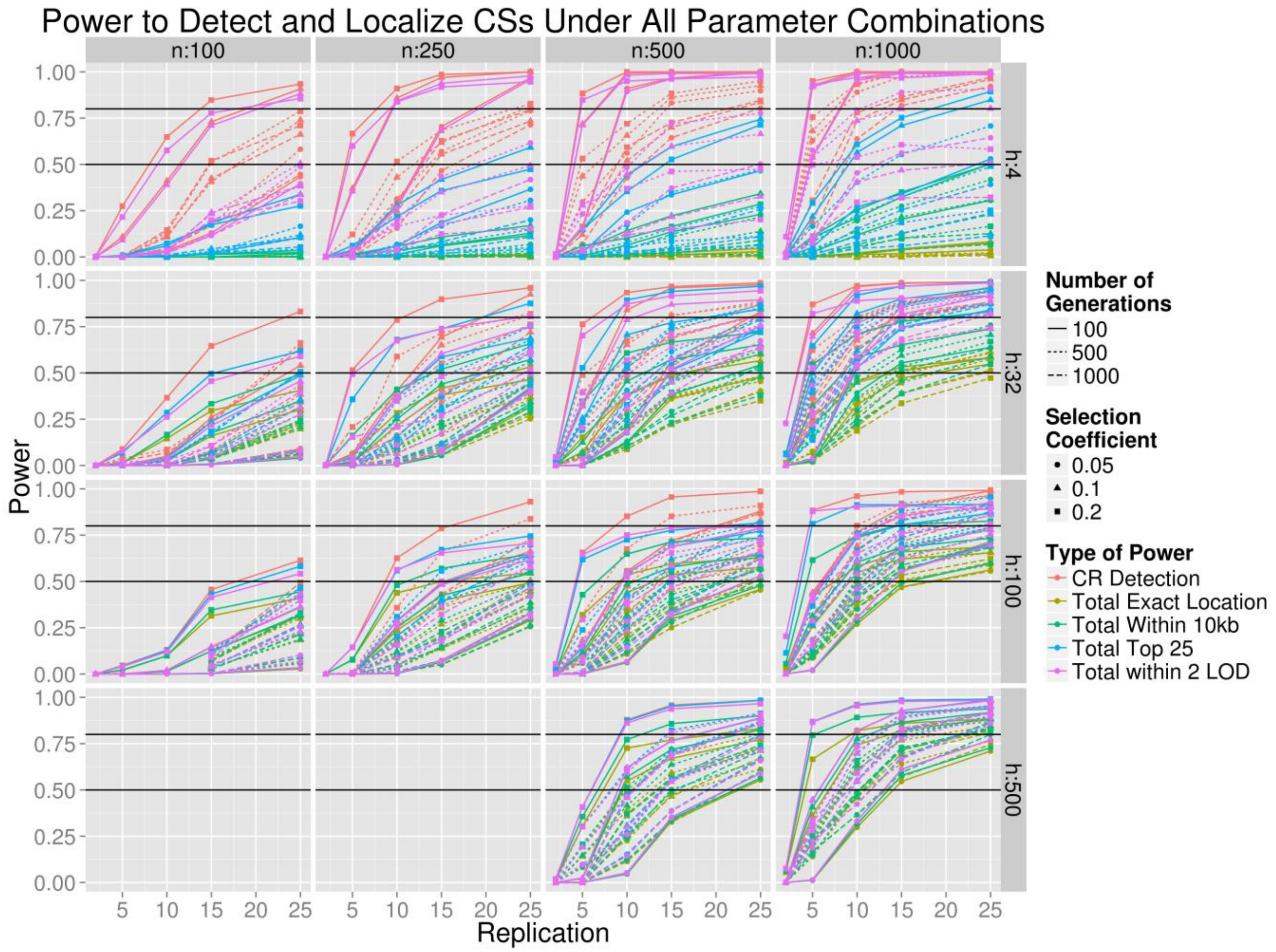

Sup. Fig. 6: The ability (total power) to detect a CS-containing region and either correctly identify the exact location of a CS or decrease the number of candidate loci to a manageable number under all Θ. In other words, the fraction of all simulations in which a region is a region contains a significant SNP and one of three methods of detecting a CS is successful: the MSM is the CS (Total Exact Location Power), the CS is within 10kb of the MSM (Total Within 10kb Power), the CS is one of the 25 most significantly diverged SNPs in the region (Total Top 25 Power), or the CS is within 2 LOD of the MSM (Total within 2 LOD power). Also shown is the CR Detection Power, which is the fraction of regions that contained at least one significant SNP. The black lines indicate 50% power and 80% power.

Supplementary Figure 7

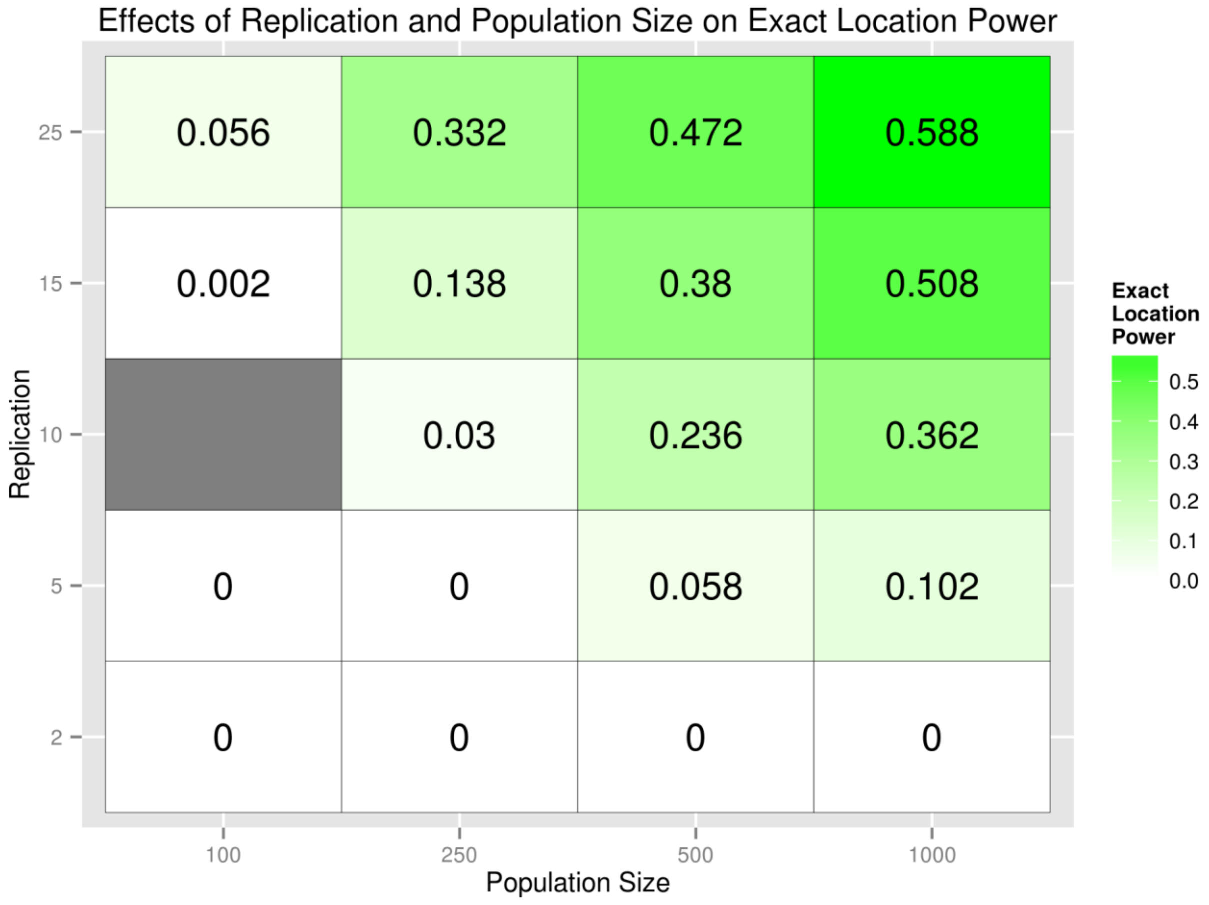

Sup. Fig. 7: Heat map depicting exact location power rate at various levels of *n* and *h*. *s*=0.05, *h*=100, *g*=500. The power where *n* = 100 and *r* = 10 is missing because none of our tested significance thresholds was strict enough to sufficiently limit false positives in that Θ.

Supplementary Figure 8

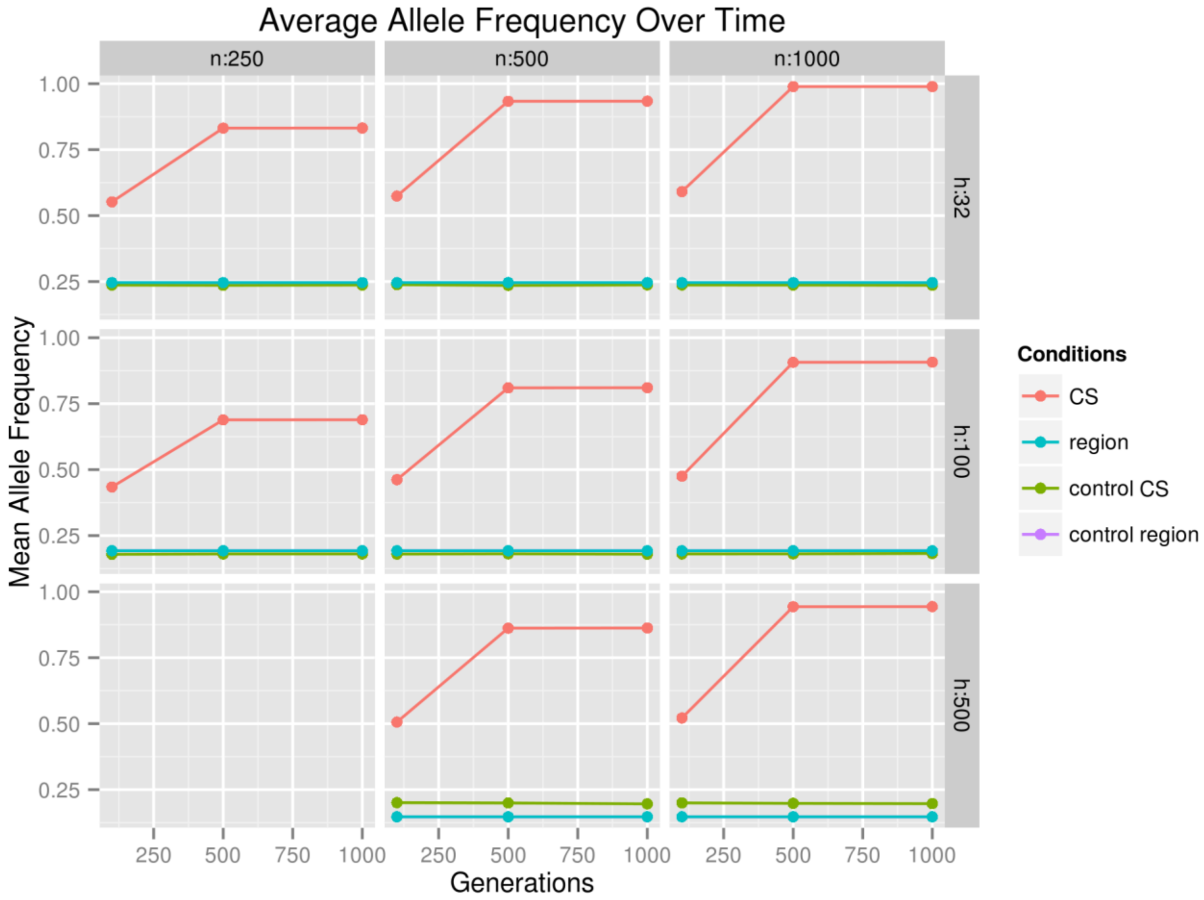

Sup. Fig. 8: Average allele frequency versus number of generations of selection. r=25, s=0.05. The red line indicates the average allele frequency of CS alleles, while the blue line indicates the average allele frequency for every SNP across regions. The green and purple lines correspond to these same values, but in the non-selected control populations. Note that this plot makes use of all available replicates for every Θ.

Supplementary Figure 9

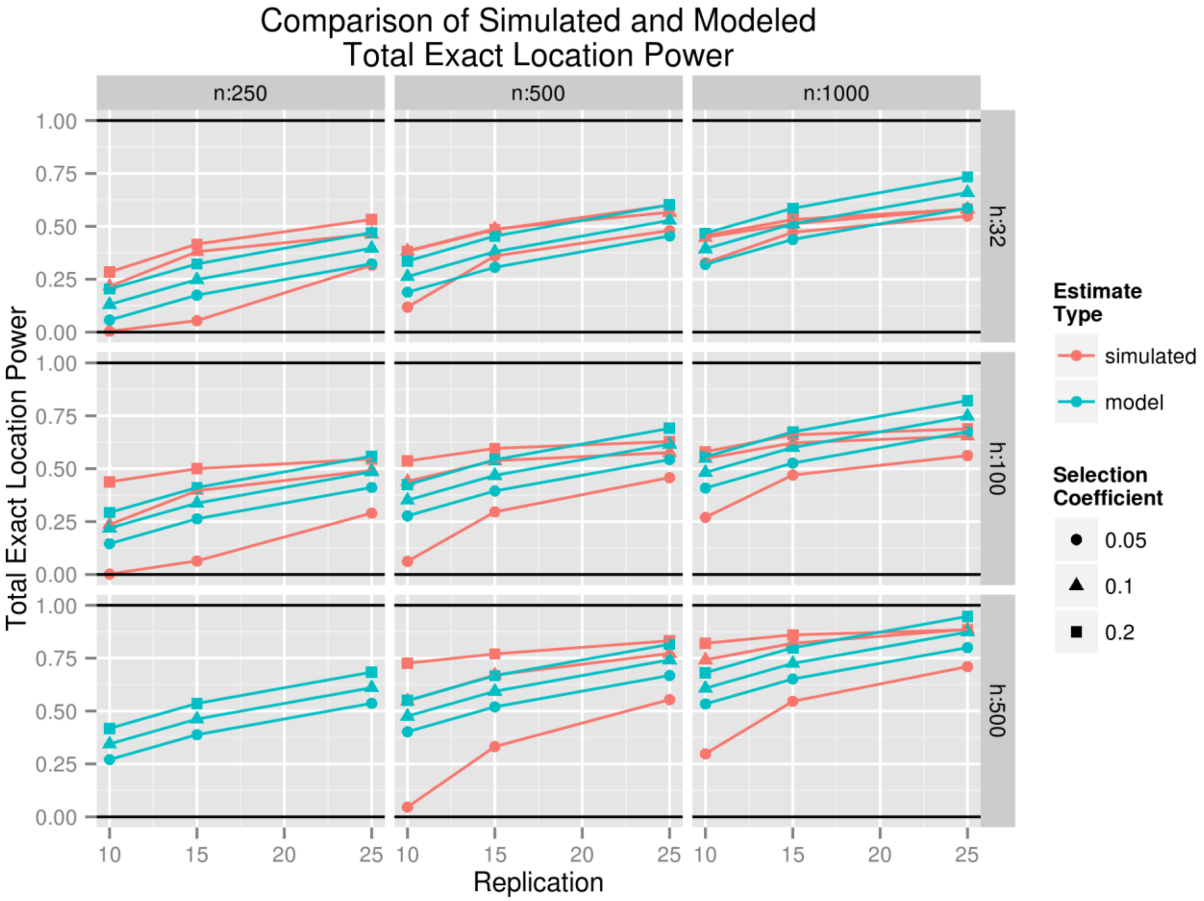

Sup. Fig. 9: A comparison of the total exact location power estimates generated by our simulations and the power estimate generated by our linear model. Only the space in which the model is relatively accurate is shown. "Simulated" estimate type refers to the total exact location power generated by our simulation. "Model" estimate type refers to the total exact location power from our mathematical model. Variation in *g* is not shown for clarity.

Supplementary Figure 10

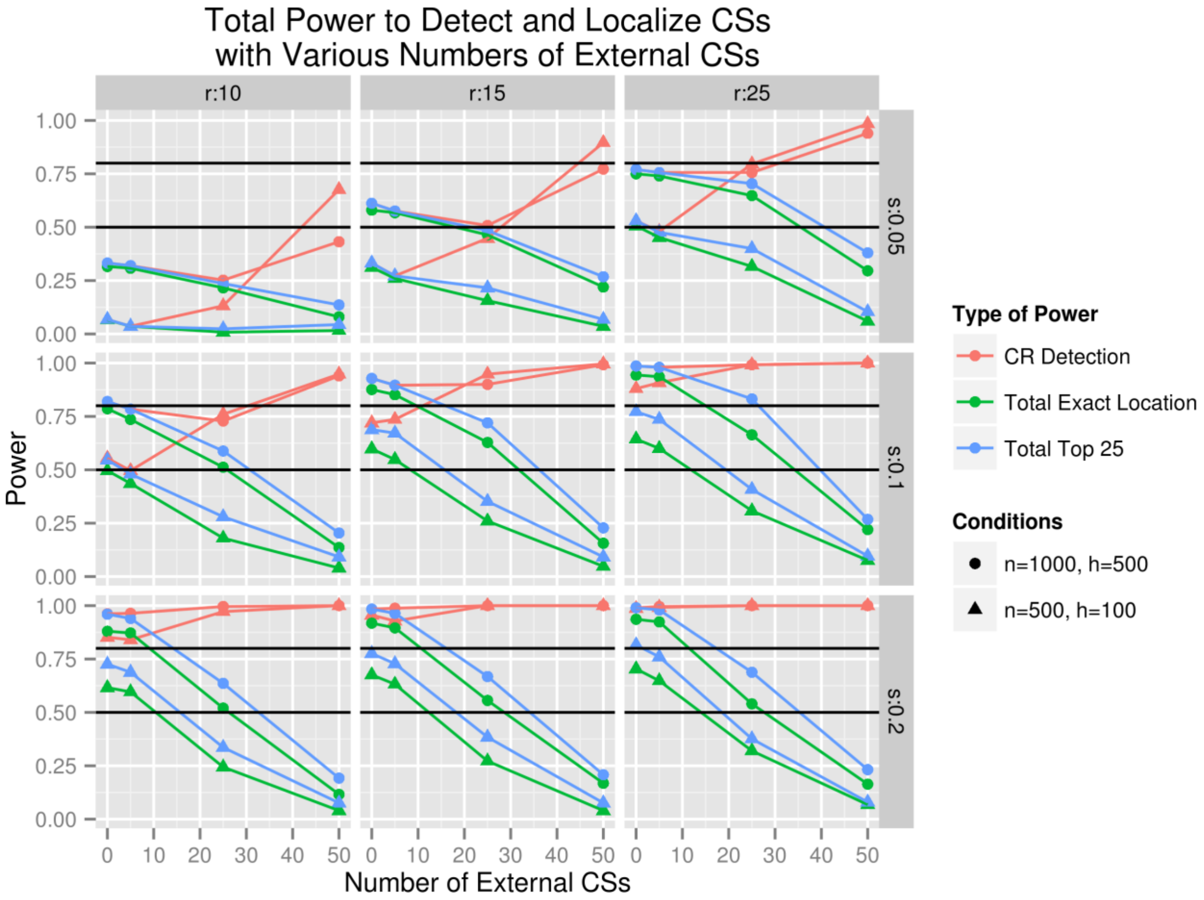

Sup. Fig. 10: A comparison of CR detection power and total power when the number of external CSs is varied. External CSs here refer to CSs that are outside of the 1Mb region surrounding the focal CS. These external CSs are randomly distributed throughout a 20Mb region and have the same selection coefficient as the focal CS.

Supplementary Figure 11

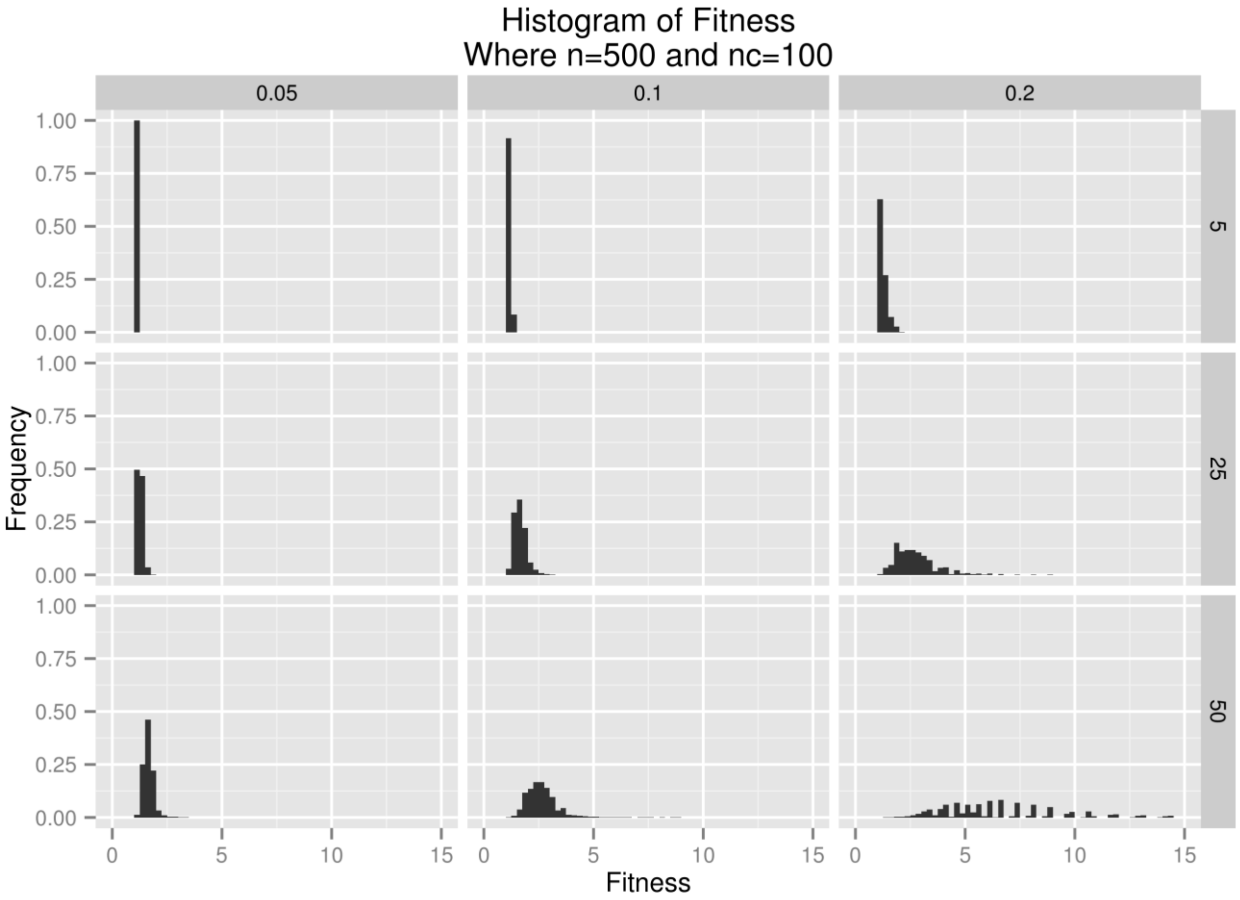

Sup. Fig. 11: This histogram illustrates the distribution of fitnesses across all simulated populations at a given Θ. All fitnesses shown here are relative to the base (mutation free) fitness, which was assigned a relative fitness of 1.

Supplementary Figure 12

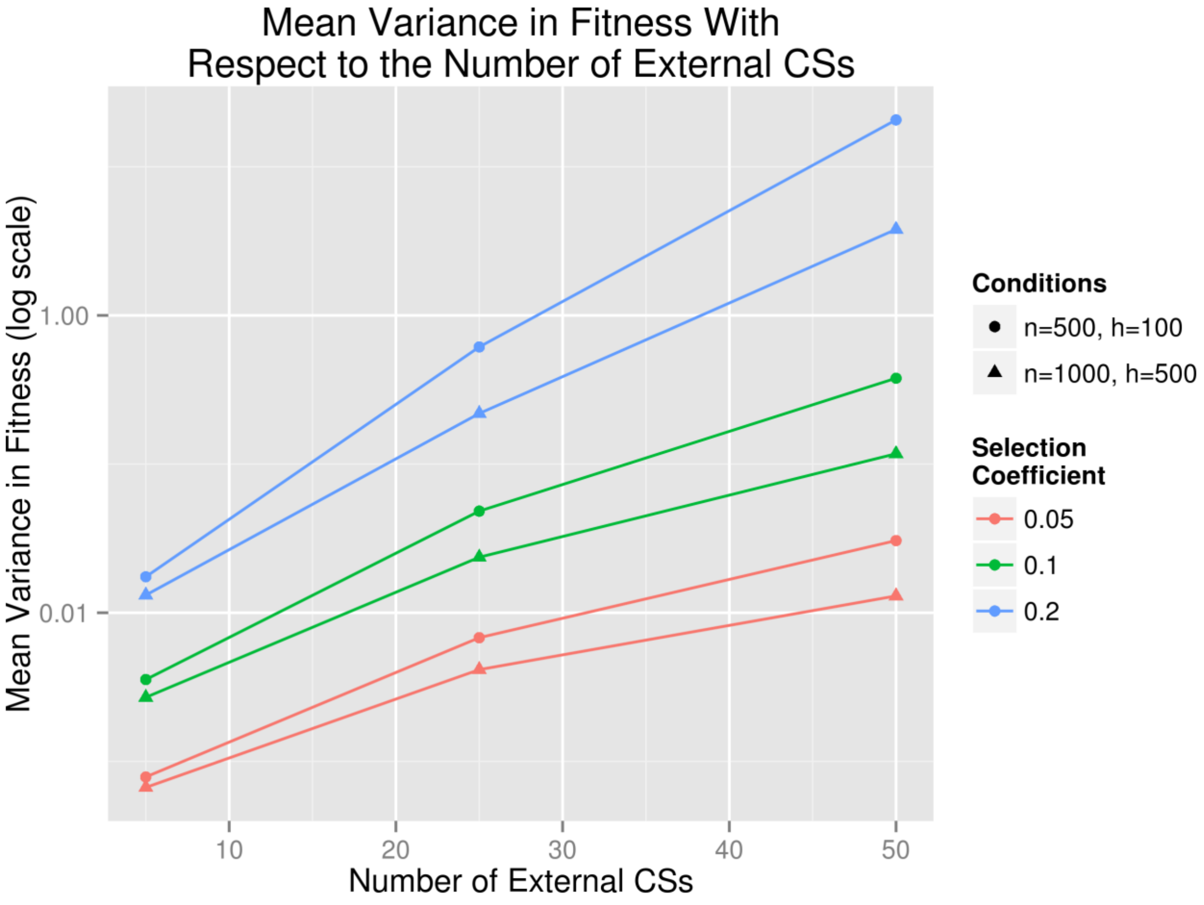

Sup. Fig. 12: This plot demonstrates the change in variance in fitness caused by the increase in the number of external CSs in a simulated chromosome. Each point represents the mean of the variances from 250 independent simulations. Note that, as these values were computed at the beginning of the forward simulation, number of generations is not taken into account.

Supplementary Figure13

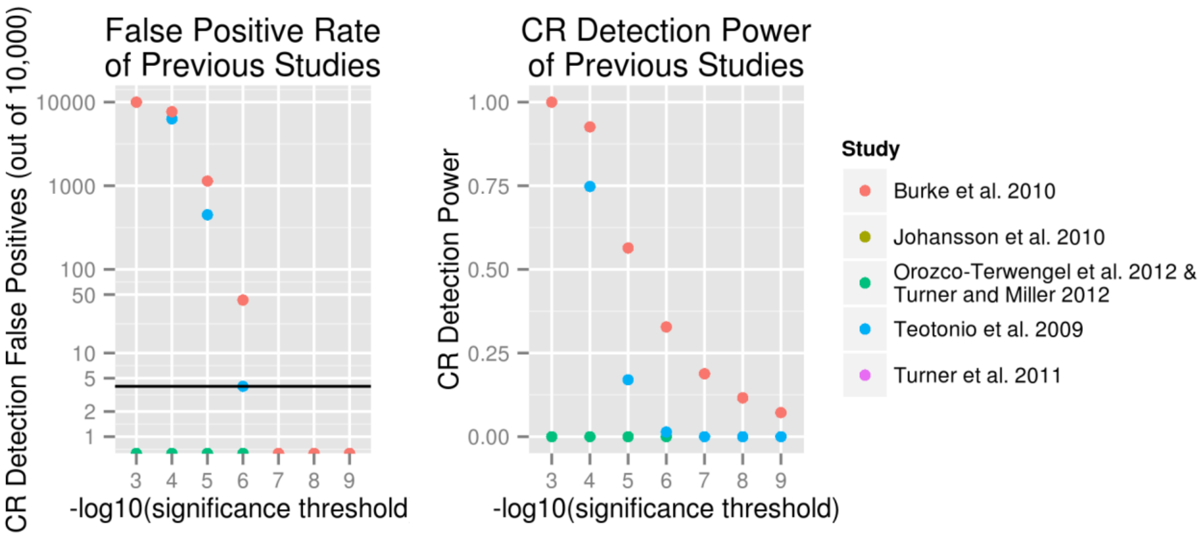

Sup. Fig. 13: CR detection power and false positive rate versus significance threshold. The Θ that most closely correspond to the experimental parameters used in existing E&R experiments are depicted. Burke et al. 2010: h = 500, n = 1000, r = 5, g = 500. Johansson et al. 2010: h = 32, n = 100, r = 2, g = 100. Orozco-Terwengel et al. 2012 and Turner and Miller 2012: h = 100, n = 1000, r = 2, g = 100. Turner et al. 2011: h = 100, n = 250, r = 2, g = 500. When a parameter value was unknown, the value that provided the highest power was chosen. Only s = 0.05 is shown. Any points that are not visible are overlapping at y = 0.
